# Patient and public involvement in healthcare: a systematic mapping review of systematic reviews – identification of current research and possible directions for future research

**DOI:** 10.1136/bmjopen-2023-083215

**Published:** 2024-09-19

**Authors:** Jana Bergholtz, Axel Wolf, Vanessa Crine, Helena Cleeve, Maria-Jose Santana, Ida Björkman

**Affiliations:** 1 Sahlgrenska Academy, University of Gothenburg Centre for Person-Centred Care, Gothenburg, Sweden; 2 Sahlgrenska Academy, University of Gothenburg Institute of Health and Care Sciences, Gothenburg, Sweden; 3 Department of Medical and Translational Biology, Umeå University, Umea, Sweden; 4 University of Gothenburg Department of Sociology and Work Science, Gothenburg, Sweden; 5 Cumming School of Medicine, University of Calgary Department of Community Health Sciences, Calgary, Alberta, Canada; 6 Patient Engagement Team, Alberta Strategy for Patient-Oriented Research (SPOR) SUPPORT Unit, Calgary, Alberta, Canada

**Keywords:** Patient Participation, Health Services, Patient-Centered Care, MEDICAL EDUCATION & TRAINING, Quality in health care, Health & safety

## Abstract

**Abstract:**

**Objectives:**

To provide an overview of patient and public involvement (PPI) in the mesolevel and macrolevel of healthcare (different from PPI in research) and identify directions for future research by mapping contexts, terminology, conceptual frameworks, measured outcomes and research gaps.

**Design:**

Mapping review of systematic reviews. A patient coresearcher (JB) was involved in all stages. A broad search strategy was applied to capture the variation in terminology.

**Data sources:**

MEDLINE, CINAHL and PsycINFO were searched from 1 January 2001 to 5 December 2022.

**Eligibility criteria:**

We included systematic reviews of empirical studies focusing on PPI in the mesolevel and macrolevel of healthcare.

**Data extraction and synthesis:**

Three independent reviewers used standardised methods to screen studies and extract data. Thematic categories were created inductively through iteration. The results were organised in narrative, visual or tabular formats.

**Results:**

4419 identified records were screened. 37 systematic reviews were eligible for inclusion. Most studies were narrative syntheses (N=26). Identified context categories were PPI for healthcare quality improvement (22%), patient safety (8%), community-based initiatives (27%), peer support (16 %) and education of healthcare professionals (27%). A wide range of terms was used to discuss PPI, with community participation being the most common. 28 reviews reported on frameworks, conceptual guidance and/or policy documents. Nine different types of outcomes were identified. The research gap pointed out most frequently is the lack of studies of robust designs that allow for replication and long-term follow-up, followed by studies on cost-effectiveness and resources needed. There is a need for consensus on the use of terminology.

**Conclusions:**

This mapping review sheds light on the evolving landscape of PPI in healthcare. To advance the field, future research should prioritise rigorous study designs, cost-effectiveness assessments and consensus-building efforts to create a more unified and impactful approach for PPI in healthcare.

**STRENGTHS AND LIMITATIONS OF THIS STUDY**

- This mapping review of systematic reviews provides a broad overview of research activity and employs a search strategy designed to capture the wide range of terminology used for patient and public involvement in healthcare.
- A patient coresearcher (JB) was included as team member and coauthor of the study.
- Mapping reviews, by design, do not allow for an in-depth description of the field, however, key data and findings are highlighted in tabular format.
- Grey literature was excluded, language was restricted to English and a second search update could not be performed due to resource constraints, potentially limiting the scope of this mapping review.
- We did not assess the quality of the 37 systematic reviews and did not check whether they included some of the same studies.

## Background

 Patient and public involvement (PPI) in healthcare has been broadly defined as ‘ways in which patients can draw on their experience and members of the public can apply their priorities to the evaluation, development, organisation and delivery of health services’.^1^ Several international and national policy documents support the principles of PPI in healthcare.^2 3^ An early example is the 1978 Declaration of Alma-Ata which stated the community is central to the development of healthcare services.^4^ In Europe, the implementation of person-centred care can be fostered with the standard EN 17398:2020 Patient involvement in healthcare—Minimum requirements for person-centred care.^5^ General arguments for PPI are based on democratic and ethical rights^6^ as well as patient empowerment.^7^

Despite the international interest in enabling healthcare systems to involve patients, family caregivers, communities and the public in decisions, major challenges for studying the impact and effects of PPI are inconsistent terminology, methods and reporting.^8 9^ Even though some of the core aspects may overlap, Modigh *et al* pointed out that it is important to distinguish between PPI in healthcare versus PPI in research in order to develop a more nuanced understanding.^9^ In their scoping review of reviews, they found many reviews reporting on the impact of patient involvement in individual care and very few reviews covering PPI at the mesolevel and macrolevel of healthcare.^9^ This is surprising, especially because this field has seen a great deal of activity in the past two decades.

Several studies also raise the lack of a theoretical basis for understanding how PPI induces improvement as a major problem for PPI in practice.^10 11^ There is a commonly expressed frustration in the field regarding the difficulty in describing how to do PPI, that is, without engaging in tokenism, so it will achieve meaningful results.^11 12^ While Greenhalgh *et al* provide a starting point for anyone who is new to PPI in research^13^—to our knowledge, such a collection of conceptual frameworks is lacking for PPI at the mesolevel and macrolevel of healthcare.

With our systematic mapping review of reviews, we aim to provide a broad overview of activities in the field of PPI at the mesolevel and macrolevel in healthcare while addressing the variation in terminology. While being attentive to the distinctions that can be drawn, we use ‘PPI’ to include patients, survivors, family members, informal caregivers, service users, community or charity representatives and citizens.

### Purpose

The overarching goal of this mapping review is to provide an overview of PPI in the mesolevel and macrolevel of healthcare and identify possible directions for future research.

Our review addresses the following research questions:

1. In which contexts is PPI conducted?
2. What are the characteristics of participants involved in PPI?
3. Which conceptualisations (terms, frameworks) of PPI are used?
4. Which outcome measures are reported for PPI?
5. What findings and research gaps can be identified?

## Method

### Patient and public involvement

A patient coresearcher (JB) was involved in all stages of the review as the first author through a collaborative partnership-focused approach based on Boote *et al*’s principles of successful consumer involvement in research.^13 14^ Partnering with a patient coresearcher influenced the whole process from research questions being asked to writing the manuscript and disseminating findings.

### Design

A mapping review aims to provide a broad overview of a research field, categorising existing literature to identify gaps from which further reviews and/or primary research can be commissioned.^15^ Mapping studies depict research activity and locations where it occurs, the flow of information and linkages.^16^

We performed a systematic mapping review of reviews; following the Preferred Reporting Items for Systematic Reviews and Meta-Analyses (PRISMA) 2020 reporting guidance^17^ as much as possible. The study protocol was preregistered on the Open Science Forum (https://osf.io/9b5j3).

The generally explored topics trace back to our research questions, which were defined in accordance with the PICo framework (Population or Problem, Interest and Context) for qualitative studies^18^ with:

- Population: patients and public of all ages involved in PPI initiatives or interventions in healthcare, not restricted to any sociodemographic characteristic or geographical region.
- Interest: the involvement of patients and the public in the mesolevel and macrolevel of healthcare.
- Context: all healthcare contexts in which PPI is implemented, not restricted to any particular healthcare service type or a particular healthcare sector.

After research questions were defined, four major steps followed: searching, screening, data extraction and analysis/thematisation. No formal quality assessment was performed.^15^

### Search and screening

A broad search strategy based on the PICo framework was used to capture the variation in the terminology used for PPI in publications (see online supplemental file 1 for search terms used for each part of PICo). Systematic electronic searches using a keyword search in the titles and abstracts were conducted by librarians of the Biomedical Library at Gothenburg University on 15 December 2021 and on 5 December 2022 within the following databases: MEDLINE via PubMed, CINAHL via EBSCO and PsycINFO via ProQUEST (see also online supplemental file 1). The search strategy was piloted against appropriate reviews to ensure that the relevant literature was identified. Search results were imported to the software Rayyan. The screening of abstracts and titles was performed independently by two reviewers (first search: JB and VC, updated search: JB and IB). Disagreements were resolved through discussion or by consulting an additional review author (AW).

#### Inclusion and exclusion criteria

Reviews were selected based on predefined eligibility criteria (see online supplemental file 1). Systematic reviews of empirical studies, which focused on the involvement of patients and the public at the mesolevel and macrolevel in healthcare, were eligible for inclusion when published in English in peer-reviewed journals. Searches were restricted to reviews published in English between 2001 and 2022 as a previous review on a similar topic^19^ searched the literature published until 2000. Other types of articles (eg, single studies, case reports, commentaries, editorials and review articles) and other types of reviews were excluded (eg, scoping, realist, rapid and umbrella) as well as systematic reviews published before 2001 or published in non-peer-reviewed journals. Systematic reviews including grey literature were excluded. Systematic reviews of studies not explicitly reporting on PPI in healthcare were excluded as well as reviews of studies on PPI in social care, due to the large differences between these domains of care. Reviews concerned with PPI in research, the engagement of participants as research subjects (eg, clinical trials) or in health technology assessment were also excluded, as these topics are more closely related to the field of medical and health research or health economics and have been extensively covered in previous reviews.

### Data extraction and thematisation

A standardised data extraction form was used to extract data in Excel (online supplemental file 1). This form was predeveloped, pilot-tested on five randomly selected studies (by JB, VC and HC) and then adapted before the overall data extraction process started. Data were extracted by JB, VC and IB. We then systematically summarised extracted data related to our research questions and organised results in narrative, visual or tabular formats to provide the reader with a clear and structured overview.

More specifically, for research questions 1 (in which contexts is PPI conducted?) and 4 (which outcome measures are reported for PPI?), extracted data were organised in related themes. Preliminary themes for context and outcome categories were formed which were refined again and again as the extracted data were re-examined in an iterative process with continuing discussion between IB and JB. For outcomes, we included any proposed or eventuating positive or negative change short term or long term.

For research question 3 (which conceptualisations (terms, frameworks) of PPI are used?), in addition to data extraction, frameworks and guidance documents cited in the reviews were tracked down to the original publication and collected. To capture the broad terminology used in the reviews, the pdf file of each article was assessed via the search function to count which term(s) and/or combination of terms were used most often to describe and discuss PPI.

## Results

### Selection and characteristics of the included reviews

**Figure 1:**
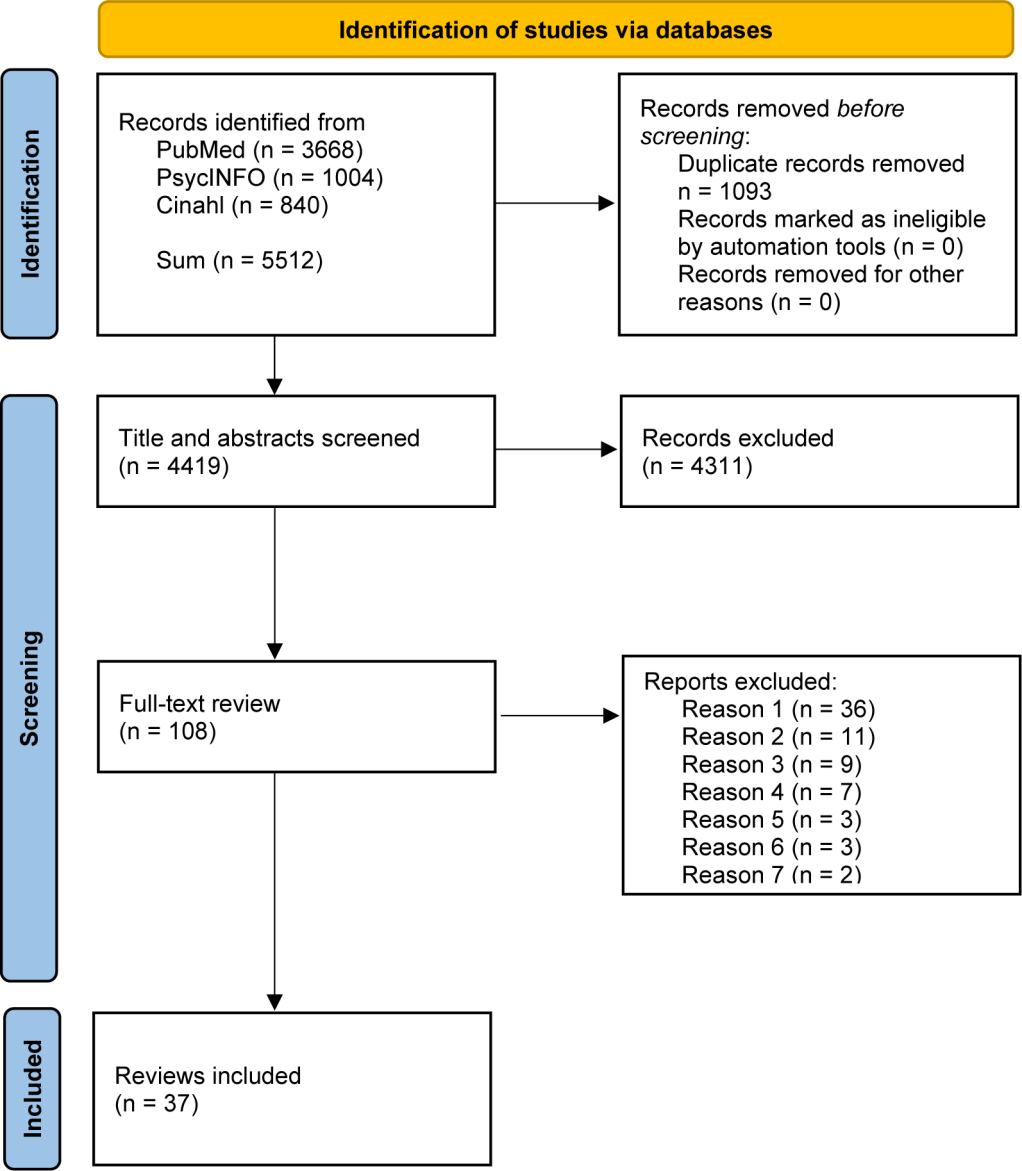
PRISMA flow diagram. Reasons for exclusion: Reason 1: Systematic review searching/including grey literature, Reason 2: Other type of article or not a systematic review of empirical studies, for example, articles including reviews, more than 50% descriptive studies or case reports or policy documents. Reason 3: Focus on patient engagement/involvement solely in their individual healthcare decisions. Reason 4: Not explicitly reporting on the involvement of patients and the public in healthcare. Reason 5: No focus on patient/public involvement. Reason 6: About PPI in research. Reason 7: No demarcation between real patients and simulated patients/actors. PPI, patient and public involvement; PRISMA, Preferred Reporting Items for Systematic Reviews and Meta-Analyses.

The PRISMA flow diagram^17^ in figure 1 presents the result of our search, detecting 5512 studies and 4419 after deduplication. After screening titles and abstracts, 108 articles were included for full-text review. 37 reviews were included as they met eligibility criteria. A list of excluded articles and reasons for exclusions can be found in online supplemental file 2.

Most reviews were narrative syntheses (N=26),^20^,^45^ followed by meta-analyses (N=6),^46^,^51^ evidence reviews (N=3)^52^,^54^ and thematic analyses (N=2).^55 56^ 11 reviews were published before 2015 and the remaining 26 after indication of growing interest. 11 reviews had an explicit focus on low or middle-income countries.^2024 30 32^,^34 46^ Two reviews focused on one country^21 28^ while the remaining 35 reviews included primary studies conducted in several countries.

A table with details of all 37 included systematic reviews can be found in online supplemental file 3. Only seven reviews were reported on PPI in the review process,^36 38 46 49 50 53 54^ see online supplemental file 4 for an assessment of the reviews using the GRIPP2 short-form reporting checklist.^57^

### Contexts for PPI in healthcare

The search yielded 37 systematic reviews that can be broadly categorised into PPI for healthcare quality improvement (22%), for improved patient safety (8%), for community-based initiatives (27%), for peer support (16 %) and for education of healthcare professionals (27%) (see table 1).

**Table 1:** Context categories for PPI in healthcare

Healthcare quality improvement(N=8)	Patient safety(N=3)	Community-based initiatives (N=10)	Peer support (N=6)	Education of healthcare professionals (N=10)
Bombard *et al* 2018^55^Danhoundo *et al* 2018^20^Evans *et al* 2010^21^Green *et al* 2020^22^Haldane *et al* 2019^23^Kesale *et al* 2022^24^Lloyd *et al* 2021^25^Moore *et al* 2019^54^	Giap and Park 2021^51^Lee *et al* 2021^26^Park and Giap 2020^27^	Banna and Bersamin 2018^28^Farnsworth *et al* 2014^53^Haldane *et al* 2020^29^Heintze *et al* 2007^30^Hoon Chuah *et al* 2018^31^Kerrigan *et al* 2013^46^Moore *et al* 2014^32^Prost *et al* 2013^47^Rass *et al* 2020^56^Sharma *et al* 2018^48^	Gaiser *et al* 2021^52^Genberg *et al* 2016^33^Pitt *et al* 2013^50^Satinsky *et al* 2021^34^Simpson and House 2002^35^Verma *et al* 2022^49^	Dijk *et al* 2020^36^Finch *et al* 2018^37^Gordon *et al* 2020^38^Happell *et al* 2014^39^Jha *et al* 2009^40^Lalani *et al* 2019^41^Murray *et al* 2022^42^Nguyen *et al* 2021^43^Reinders *et al* 2011^44^Scott *et al* 2020^45^

PPIpatient and public involvement

15 reviews did not focus on a diagnosis/health issue but on the quality of healthcare services in general, or education of healthcare professionals.^2024 26 27 36^,^45 51^ Eight reviews reported on a mix of health issues/diagnoses/health risks.^2123 25 28 32 32 54^,^56^ Four reviews focused solely on mental health or substance abuse.^34 35 50 52^ Three reviews focused on a specific infectious disease, namely HIV^33 46^ and dengue fever.^30^

Mental health and/or substance abuse was covered by 10 reviews,^2225 29 34 35 50 52 54^,^56^ non-communicable diseases (excluding mental disorders) N=10,^21^,^2325 28 29 49 54^ infectious disease N=8,^23 30 32 32 33 46 55 56^ women and child health N=8^2532 47 48 53^,^56^ and healthy living N=6.^21 23 28 31 54 55^ See online supplemental file 5 for health issues covered by a number of reviews.

**Figure 2:**
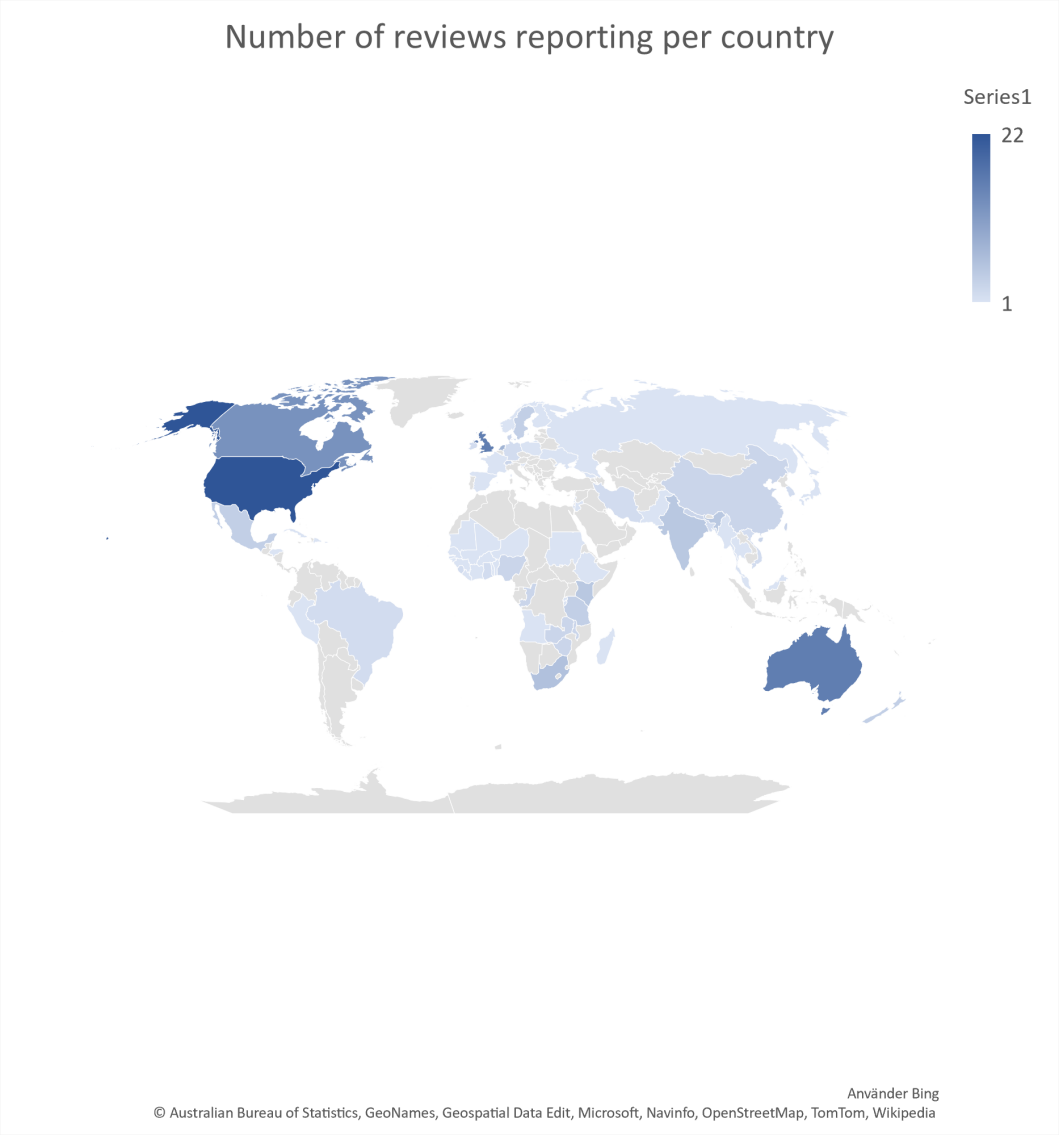
World map produced in Excel showing the number of reviews reporting from each continent.

33 reviews reported primary studies from 73 different countries from all continents while 4 reviews did not report the country for the primary studies.^27 37 39 53^ Figure 2 shows a world map indicating the number of reviews reporting per country. Additional information can be found in online supplemental file 5 (the number of reviews reporting from each continent sorted by context categories).

### Characteristics of participants

In general, age, gender, ethnicity, educational levels or socioeconomic factors were not reported on an aggregated level. However, some reviews focused on specific population characteristics including sex workers,^32 46^ women in rural low-resource settings^47^ and people affected by armed conflict.^56^

### Conceptualisations of PPI in healthcare

#### Terminology

Online supplemental file 6 shows the wide range of terms used in the 37 systematic reviews to discuss PPI. Notably, combinations of terms are used indicating who is being involved (eg, patient, user, service user, health service user, consumer, patient and family, patient and public, citizen, public, community) and how they are being involved (eg, feedback, participation, engagement, involvement, empowerment, mobilisation). In some cases, the word interventions are added (eg, patient feedback interventions, community empowerment interventions). The most common combination of terms in the included reviews is community participation occurring in the context of both, community-based initiatives and healthcare quality improvement.

Some authors decided to avoid the complications arising from using terms with similar meaning and used instead a more general framing such as ‘participatory approaches’,^21^ ‘participatory methods’^54^ or ‘participatory learning and action’.^47^

#### Frameworks, conceptual guidance and policy documents for PPI

A majority of the reviews (n=28) reported on frameworks, conceptual guidance and/or policy documents for PPI.^20^,^2224^ 13 systematic reviews applied PPI frameworks to analyse, present and/or discuss their results.^2225^,^27 31 32 36 38 42 51 53^ Table 2 provides an overview of all frameworks, conceptual guidance and policy documents cited in the five context categories for PPI.

**Table 2:** Frameworks, conceptual guidance and policy documents for PPI in healthcare

Healthcare quality improvement	Patient safety	Community-based initiatives	Peer support	Education of healthcare professionals
**Accra Agenda for Action 2008**^74^ cited by Danhoundo *et al* 2018^20^**Alma Ata, WHO 1978**^4^cited by Kesale *et al* 2022^24^**Arnstein’s ladder of citizen participation**^62^cited by Bombard *et al* 2018^55^**Bate and Robert’s framework**^75^ discussed by Bombard *et al* 2018^55^**Carman’s framework**^76^discussed by Bombard *et al* 2018^55^**CeHRes Roadmap**^77^discussed by Moore *et al* 2019^54^**Decentralisation**^78^cited by Kesale *et al* 2022^24^**Experience-based co-design (EBCD) toolkit**^22^discussed by Green *et al* 2020^22^**IAP2 Spectrum**^63^discussed by Lloyd *et al* 2021^25^**Community Engagement, NICE Guidance**^79^cited by Evans *et al* 2010^21^**Paris Declaration 2005**^74^cited by Danhoundo *et al* 2018^20^**Social accountability**^80^cited by Danhoundo *et al* 2018^20^	**Carman’s framework**^76^discussed by Giap and Park 2021^51^ and Park and Giap 2020^27^**Canadian guide for engaging patients in patient safety**^81^discussed by Park and Giap 2020^27^**Higgins** ***et al*** **2017**^82^cited by Giap and Park 2021^51^ and Park and Giap 2020^27^**Longtin** ***et al*** **2010**^83^cited by Park and Giap 2020^27^ and Lee *et al* 2021^26^**NHS Framework for Patient Engagement in Patient Safety**^84^discussed by Lee *et al* 2021^26^**WHO Framework on Patient and Family Engagement**^85^cited by Park and Giap 2020^27^ and Lee *et al* 2021^26^	**Arnstein’s ladder of citizen participation**^62^cited by Haldane *et al* 2020^29^ and Hoon Chuah *et al* 2018^31^**Ashodaya’s model**^86^discussed by Moore *et al* 2014^32^**Community Engagement Continuum**^64^discussed by Farnsworth *et al* 2014^53^**Integrated Model of Communication for Social Change (IMCSC) framework**^87^discussed by Farnsworth *et al* 2014^53^**Popay’s framework**^65^ cited by Haldane *et al* 2020^29^ and discussed by Hoon Chuah *et al* 2018^31^**Rosato** ***et al*** **2008**^88^cited by Prost *et al* 2013^47^**Stone 1992**^89^cited by Sharma *et al* 2018^48^**Wallerstein 1992**^90^ cited by Kerrigan *et al* 2013^46^, Prost *et al* 2013^47^ and Moore *et al* 2014^32^**WHO’s wheel of participation**^66^cited by Hoon Chuah *et al* 2018^31^ and Haldane *et al* 2020^29^	**Caro and Fischer 2010**^91^cited by Verma *et al* 2022^49^**Dennis 2003**^92^cited by Satinsky *et al* 2021^34^**National Service Framework for Mental Health (UK)**^93^cited by Simpson and House 2002^35^**Recovery Support Tools and Resources, Guidance by Substance Abuse and Mental Health Services Administration (USA)**^69^cited by Gaiser *et al* 2021^52^ and Satinsky *et al* 2021^34^**Simoni** ***et al*** **2011**^94^cited by Genberg *et al* 2016^33^**Voices in partnership: involving users and carers in commissioning and delivering mental health services**^95^cited by Simpson and House 2002^35^	**Arnstein’s ladder of citizen participation**^62^cited by Gordon *et al* 2020^38^**Cambridge Framework by Spencer** ***et al*** **2000**^96^described by Gordon *et al* 2020^38^**Lathlean** ***et al*** **2006**^97^cited by Happell *et al* 2014^39^**Patient and public involvement in undergraduate medical education, General Medical Council**^98^cited by Dijk *et al* 2020^36^**Rowland et al 2019**^99^cited by Murray *et al* 2022^42^**Tew** ***et al*** **2004**^67^cited by Gordon *et al* 2020^38^**Towle** ***et al*** **2010**^68^ discussed by Dijk *et al* 2020^36^ and Gordon *et al* 2020^38^, cited by Nguyen *et al* 2021^43^**Tritter’s framework**^1^cited by Lalani *et al* 2019^41^

PPIpatient and public involvement

### Outcome measures for PPI in healthcare

We classified the reviews according to nine different types of outcomes; community outcomes, costs, discrete products, governance outcomes, health correlates, health outcomes, participants’ perceptions and knowledge, service outcomes, service providers’ perceptions and knowledge (see table 3).

**Table 3:** Outcomes reported in reviews

Outcome	Reviews reporting
Community outcomes	Danhoundo *et al* 2018,^20^ Farnsworth *et al* 2014,^53^ Haldane *et al* 2019,^23^ Hoon Chuah *et al* 2018,^31^ Moore *et al* 2014,^32^ Rass *et al* 2020^56^
Costs	Farnsworth *et al* 2014,^53^ Green *et al* 2020,^22^ Prost *et al* 2013^47^
Discrete products	Bombard *et al* 2018,^55^ Danhoundo *et al* 2018,^20^ Lloyd *et al* 2021,^25^ Moore *et al* 2019,^54^ Murray *et al* 2022^42^
Governance outcomes	Bombard *et al* 2018,^55^ Danhoundo *et al* 2018,^20^ Haldane *et al* 2019,^23^ Kesale *et al* 2022,^24^ Lloyd *et al* 2021^25^
Health correlate outcomes	Danhoundo *et al* 2018,^20^ Pitt *et al* 2013,^50^ Simpson and House 2002^35^
Health outcomes	Banna and Bersamin 2018,^28^ Danhoundo *et al* 2018,^20^ Evans *et al* 2010,^21^ Farnsworth *et al* 2014,^53^ Gaiser *et al* 2021,^52^ Genberg *et al* 2016,^33^ Giap and Park 2021,^51^ Haldane *et al* 2019,^23^ Heintze *et al* 2007,^30^ Hoon Chuah *et al* 2018,^31^ Kerrigan *et al* 2013,^46^ Lee *et al* 2021,^26^ Moore *et al* 2014,^32^ Park and Giap 2020,^27^ Pitt *et al* 2013,^50^ Prost *et al* 2013,^47^ Satinsky *et al* 2021,^34^ Sharma *et al* 2018,^48^ Verma *et al* 2022^49^
Participants perceptions and knowledge	Banna and Bersamin 2018,^28^ Danhoundo *et al* 2018,^20^ Evans *et al* 2010,^21^ Giap and Park 2021,^27^ Green *et al* 2020,^22^ Haldane *et al* 2019,^23^ Haldane *et al* 2020,^29^ Happell *et al* 2014,^39^ Jha *et al* 2009,^40^ Lee *et al* 2021,^26^ Nguyen *et al* 2021,^43^ Park and Giap 2020,^27^ Pitt *et al* 2013^50^
Service outcomes	Bombard *et al* 2018,^55^ Danhoundo *et al* 2018,^20^ Farnsworth *et al* 2014,^53^ Giap and Park 2021,^51^ Green *et al* 2020,^22^ Haldane *et al* 2020,^29^ Hoon Chuah *et al* 2018,^31^ Lalani *et al* 2019,^41^ Lee *et al* 2021,^26^ Lloyd *et al* 2021,^25^ Park and Giap 2020,^27^ Pitt *et al* 2013,^50^ Rass *et al* 2020,^56^ Sharma *et al* 2018^48^
Service providers perceptions and knowledge	Danhoundo *et al* 2018,^20^ Dijk *et al* 2020,^36^ Evans *et al* 2010,^21^ Finch *et al* 2018,^37^ Gordon *et al* 2020,^38^ Haldane *et al* 2019,^23^ Haldane *et al* 2020,^29^ Happell *et al* 2014,^39^ Jha *et al* 2009,^40^ Lalani *et al* 2019,^41^ Nguyen *et al* 2021,^43^ Park and Giap 2020,^27^ Reinders *et al* 2011,^44^ Scott *et al* 2020^45^

Community outcomes included attitudes, acceptability or empowerment of communities and intervention coverage. Costs included comparisons of methods for PPI and cost-effectiveness. Discrete products were educational packages, tools, policy documents, competency frameworks and eHealth resources. Examples of health outcomes include changes in the physical and mental health status of the individual as well as health-related behaviours. Examples of this category are body mass index (BMI), quality of life, hospital admission, incidence of infectious disease and adherence to treatment. Health correlates include infrastructure and sanitation ratings as well as employment. Examples of governance outcomes include organisational culture change and patient representation on boards or community priority settings. Participants’ perceptions and knowledge include those of service users, patients and their families as well as others involved in PPI such as peers. Examples of service outcomes include access, availability, use, quality, development of new services and care pathways, adverse events, length of hospital stay and work environment. Service providers’ perceptions and knowledge also include those of students.

### Findings and identified research gaps

In the category of community-based initiatives, there was one meta-analysis showing that PPI was non-significantly associated with a reduction of maternal and neonatal mortality.^47^ In the area of patient safety one meta-analysis showed that PPI significantly reduced adverse events, decreased the length of stay, increased patient safety experiences and improved patient satisfaction.^51^ For peer support one meta-analysis found that there were statistically significant changes in haemoglobin A1c after the interventions. However, there were no significant changes in low-density lipoprotein, BMI, systolic blood pressure and health-related quality of life).^49^ No meta-analysis was identified in the category of education of healthcare professionals but findings show that patients were engaged in various educational settings within and outside the hospital^36^ mainly in the role of teacher^36^ and more seldom as formative assessor^36 40^ or in curriculum development.^40^ PPI improved students’ clinical and communication skills,^37 38^ facilitated holistic and humanistic qualities and a better understanding of person-centred care.^38 44^ No meta-analysis was found in the category healthcare quality improvement but findings show that the level of engagement appears to influence the outcomes.^55^ Evans *et al* report that very little evidence exists of participatory approaches having any noteworthy impact on health or social outcomes^21^ while another review reports positive outcomes on organisational, community and individual level.^23^

The research gap pointed out most frequently is the lack of studies of robust designs that allow for replication and long-term follow-up,^2021 23 25 26 29 31^,^36 40 43 50 52^ followed by studies on cost-effectiveness and resources needed.^23 25 29 30 38 40 47 52^ Other research gaps identified are the need for conceptual, terminology and guideline consensus within the field.^22 27 36 51 54 55^ Two reviews point out certain target groups missing from the available literature, being indigenous youth^28^ and males^48^ while two reviews wish for more qualitative data on how patients themselves experience the involvement process or how they are meaningfully engaged.^44 55^

## Discussion

The findings of this mapping review of systematic reviews provide valuable insights into the state of PPI in the mesolevel and macrolevel of healthcare. The results indicate that most systematic reviews in this area are narrative syntheses, followed by meta-analyses, evidence reviews and thematic analyses. This suggests that while there is a growing interest in PPI, the evidence base is diverse, which challenges when trying to draw comprehensive conclusions.

A notable shift in the publication of systematic reviews can be seen, with a growing interest in PPI in healthcare after 2015. This trend reflects the increasing recognition of the importance of involving patients and the public in healthcare decision-making processes.^2 10 58^ Several areas for PPI were identified showing that it is not limited to a single context; rather, it is employed across healthcare quality improvement, patient safety, community-based initiatives, peer support and education of healthcare professionals. This diversity underscores the adaptability and relevance of PPI in addressing various healthcare challenges.

The review also highlights the global reach of PPI, with primary studies included from 73 different countries across all continents. This international scope reflects the universal applicability and importance of involving patients and the public in healthcare decision-making. PPI in healthcare quality improvement, patient safety and the education of healthcare professionals mostly occurs in high-income countries. Community-based initiatives are mainly conducted in low-income/middle-income countries and primarily concern infectious disease control and women/child health. Peer support, on the other hand, appears to occur predominantly in mental health/substance abuse in North America and Europe.

The data reveal a wide range of terms used to describe and discuss PPI at the mesolevel and macrolevel of healthcare. This diversity in terminology can be both enriching and challenging, as it reflects the evolving nature of the field.^10 58^ The variation in terms used to describe PPI activities and interventions in healthcare confirms previous findings and underlines current discussions of a ‘conceptual muddle’ that might hamper the implementation of PPI.^5559^,^61^ Thus, standardising the language could enhance clarity and communication in this area.

Many systematic reviews reported on frameworks, conceptual guidance and policy documents for PPI. This indicates that researchers and practitioners are drawing on established frameworks to guide their PPI efforts, which can contribute to consistency and best practices. Several systematic reviews refer to Arnstein’s ladder of citizen participation^62^ and some of the frameworks that are being applied for PPI in healthcare quality improvement, community-based initiatives and the education of healthcare professionals have clearly evolved from Arnstein’s ladder: the IAP2 spectrum,^63^ the Community Engagement Continuum,^64^ Popay’s model,^65^ WHO’s wheel of participation^66^ and Tew’s ladder.^67^ Most advancements and more detailed discussions of conceptual frameworks can be observed in systematic reviews for PPI in the education of healthcare professionals, where, for example, Gordon *et al*^38^ suggested an amendment to the use of Towle’s framework^68^ and Dijk *et al*^36^ defined new roles that could not be ascribed to a specific level in Towle *et al*’s framework.^68^ Roles for patient representatives are thus well defined in education.^36 38 68^ In peer support,^52 69^ roles are naturally more specific, but in healthcare quality improvement and community-based initiatives, roles could be better defined, see, for example, Bombard *et al*^55^ and Haldane *et al*.^29^ Only three systematic reviews identified theories and frameworks used in each included study.^31 53 54^ Hoon Chuah *et al*^31^ found that most single studies were undertheorised. Furthermore, Rass *et al*^56^ point out that there is limited critical engagement with concepts of participation in the context of crisis management.

Nine types of outcomes of PPI were identified, ranging from community outcomes to service providers’ perceptions and knowledge. This comprehensive approach to assessing the impact of PPI reflects the multifaceted nature of its influence on healthcare but this inconsistency in reporting makes it difficult to compare study results and perform meta-analyses. As for all complex interventions, outcomes must be carefully chosen in the light of the interventions purpose. To be able to choose the right outcomes, there must be an understanding and/or theory of the causal chains for why one can expect an intervention to lead to a specific outcome.^70^ Outcomes chosen assessing the impact and effectiveness of PPI also varies in relation to why one wishes to engage in such practices. Thus, what one person considers a successful involvement may differ from another’s perspective. For example, PPI is a highly valued democratic right building on the idea that those who are governed are supposed to have influence on the governance.^62^ From such a perspective, outcome for PPI chosen is empowerment of participants, partnership and mutual learning among stakeholders, local capacity building, long-term commitment and actual change of implementation sites.^71^ If involvement is a right it can be argued that it is worth doing regardless of impact but, as Staley 2015 argues in the case of PPI in research, we still need to ask what difference it makes and which is the best way to do it.^72^

To measure the effects and impact of PPI, it is also crucial to consider the complex–interplay of various factors leading to a certain outcome.^11^ As already pointed out in the Alma Ata declaration, every community might have specific local challenges and needs, meaning there is no one-size-fits-all for PPI. Again, the findings from specific categories of PPI contexts underscore the heterogeneity of results. While some areas, such as patient safety, show significant positive outcomes, others require more robust evidence.

The predominant research gap for future research in this field is the scarcity of robustly designed studies that are replicable and incorporate long-term follow-up. This concern has been highlighted across a broad range of literature sources. One crucial aspect is to define and describe the components of PPI in the study design, as the lack of consensus on concepts, terminology and guidelines within this field, underscores a critical need for standardisation and clarity. Using the European standard (EN 17398:2020) could be one step in defining and describing the components needed for minimum requirements of patient involvement in mesolevel and macrolevel healthcare.^5^ Additionally, there is a notable need for research focusing on the cost-effectiveness and resource allocation in this domain, as indicated by various studies. The existing literature seems to inadequately address the aspects of equality and equity in PPI, especially neglecting specific demographic groups such as indigenous youth and males. This oversight signals a pressing requirement for research that is more inclusive and representative of diverse populations. Lastly, there is a call for increased qualitative research that delves into patients’ personal experiences with involvement processes and how they perceive their engagement as meaningful, and its connection to person-centredness. This comprehensive approach to identifying research gaps is essential for advancing the field in a balanced and inclusive manner.

Our mapping review of systematic reviews complements recently published studies in several ways. Usher and Denis explored storylines in the PPI literature in a meta-narrative review to better understand persistent difficulties in the transformation of healthcare systems.^10^ They concluded that developments across microlevel, mesolevel and macrolevel need to be captured to see how they support one another to drive, enable and sustain change. The systematic review of reviews by Ocloo *et al* explored theory, barriers and enablers for PPI across health, social care and patient safety^73^ and found that the development of theory-driven approaches is a neglected area. Greenhalgh *et al* reported on frameworks for PPI in research,^13^ whereas we cover frameworks and conceptual guidance for PPI in the mesolevel and macrolevel of healthcare (see table 2) which can be helpful for those who are new to engaging patients and the public.

### Strengths and limitations

This study benefited from the inclusion of a patient coresearcher (JB) as a team member and author, providing a patient perspective throughout the whole process. We employed a comprehensive search strategy, allowing for a wide exploration of the terminology used in publications related to PPI in healthcare. A rigorous screening process was conducted, and the careful selection process enhanced the reliability and relevance of the included reviews. A limitation is that the language was restricted to English, which may introduce language bias where relevant research published in other languages may have been excluded, potentially limiting the scope of the mapping review. Also, grey literature was excluded, and such can sometimes contain valuable insights not found in peer-reviewed journals, and its exclusion may lead to the omission of relevant information. The study protocol was registered on the Open Science Forum, providing transparency about the research process and facilitating reproducibility. The method for systematic mapping review was chosen as this is useful for identifying patterns in a very large body of literature. Mapping reviews, by design, do not typically include quality assessment or statistical analyses of the included reviews.^15^ While this approach provides a broad overview of research activity, it does not evaluate the methodological quality or rigour of the reviews themselves and does not allow for an in-depth description of the field. However, a strength of our mapping review is that key data and findings are displayed in tabular format.

We did not check whether reviews included the same references. Some reviews studied similar topics and may therefore have included some of the same studies.

## Conclusion

In conclusion, this systematic mapping review sheds light on the evolving landscape of PPI in the mesolevel and macrolevel of healthcare. The diversity in systematic review types, contexts and terminology highlights the complexity of the field. While there is evidence of positive outcomes in certain areas, several research gaps remain to be addressed. To advance the field of PPI, future research should prioritise rigorous study designs, cost-effectiveness assessments and consensus-building efforts to create a more unified and impactful approach to involving patients and the public in healthcare decision-making. Overall, this underscores the importance of continued exploration and development of PPI practices in the mesolevel and macrolevel of healthcare to enhance the relevance, quality, safety and effectiveness of healthcare services worldwide.

## supplementary material

10.1136/bmjopen-2023-083215 online supplemental file 1

10.1136/bmjopen-2023-083215 online supplemental file 2

10.1136/bmjopen-2023-083215 online supplemental file 3

10.1136/bmjopen-2023-083215 online supplemental file 4

10.1136/bmjopen-2023-083215 online supplemental file 5

10.1136/bmjopen-2023-083215 online supplemental file 6

## Data Availability

Data are available on reasonable request. All data relevant to the study are included in the article or uploaded as online supplemental information.
