# Supplementary material for: Patient and public involvement in healthcare: a systematic mapping review of systematic reviews – identification of current research and possible directions for future research
Source: BMJ Open. 2024 Sep 19;14(9):e083215. doi: 10.1136/bmjopen-2023-083215 (PMC11418490; doi:10.1136/bmjopen-2023-083215)
Supplement: online supplemental file 1 [file bmjopen-14-9-s001.pdf]

Supplement 1.

Search Strategy

PICo framework, operationalization, and search terms		
	**Operationalization**	**Search terms**
**Population**	Patients and the public	Advocate OR caregiver OR client OR community OR consumer OR family OR lay OR patient OR patient representatives OR public OR stakeholders
**Interest**	Involvement (e.g., concepts, implementations, methods, outcomes)	Activation OR activism OR advocacy OR empowerment OR engage OR engagement OR engaging OR input OR involvement OR involving OR participation OR health OR shared decision making
**Context**	Healthcare on the macro-, meso- and micro- levels worldwide	No specific term but a downstream selection

Box 1: Search strategy for Medline

("public involvement"[tiab] OR "family involvement"[tiab] OR "public engagement"[tiab] OR "engaging stakeholders"[tiab] OR "shared decision making"[tiab] OR "Decision Making, Shared"[mesh] OR "community participation"[tiab] OR "stakeholder participation"[tiab] OR "stakeholder participation"[mesh] OR "community involvement"[tiab] OR "client participation"[tiab] OR "consumer engagement"[tiab] OR "consumer participation"[tiab] OR "consumer involvement"[tiab] OR "lay participation"[tiab] OR "lay engagement"[tiab] OR "lay involvement"[tiab] OR “public and patient involvement"[tiab] OR "patient involvement"[tiab] OR "patient and public involvement"[tiab] OR "involving patients"[tiab] OR "caregivers involvement"[tiab] OR "patient engagement"[tiab] OR "engaging patients"[tiab] OR "engage patients"[tiab] OR "patient participation"[tiab] OR "patient participation"[mesh] OR "patient engagement"[tiab] OR "patient advocacy"[tiab] OR "patient advocate"[tiab] OR "patient representatives"[tiab] OR "patient empowerment"[tiab] OR "health activism"[tiab] OR "health advocacy"[tiab] OR "patient input"[tiab] OR "patient activation"[tiab]) AND ((Search* AND (medline OR pubmed OR cinahl OR scopus OR embase)) OR "systematic"[filter] OR "meta-analysis"[pt] OR "meta-analysis as topic"[mh] OR meta analy*[tw] OR metanaly*[tw] OR metaanaly*[tw] OR met analy*[tw] OR "systematic review"[pt] OR "systematic reviews as topic"[mh] OR systematic review*[tiab] OR systematic review[ti] OR systematic literature review[ti] OR systematic scoping review[ti] OR systematic narrative review[ti] OR systematic qualitative review[ti] OR systematic evidence review[ti] OR systematic quantitative review[ti] OR systematic meta-review[ti] OR systematic critical review[ti] OR systematic mixed studies review[ti] OR systematic mapping review[ti] OR systematic cochrane review[ti] OR systematic search and review[ti] OR systematic integrative review[ti])

Box 2: Search strategy for Cinahl

((TI "public involvement" OR AB "public involvement") OR (TI "family involvement" OR AB "family involvement") OR (TI "public engagement" OR AB "public engagement") OR (TI "engaging stakeholders" OR AB "engaging stakeholders") OR (TI "shared decision making" OR AB "shared decision making") OR (MH "Decision Making, Shared+”) OR (MH "Decision Making, Patient+") OR (TI "community participation" OR AB "community participation") OR (TI "stakeholder participation" OR AB "stakeholder participation") OR (MH "stakeholder participation+”) OR (TI "community involvement" OR AB "community involvement") OR (TI "client participation" OR AB "client participation") OR (TI "consumer engagement" OR AB "consumer engagement") OR (TI "consumer participation" OR AB "consumer participation") OR (TI "consumer involvement" OR AB "consumer involvement") OR (TI "lay participation" OR AB "lay participation") OR (TI "lay engagement" OR AB "lay engagement") OR (TI "lay involvement" OR AB "lay involvement") OR (TI "public and patient involvement" OR AB "public and patient involvement") OR (TI "patient involvement" OR AB "patient involvement") OR (TI "patient and public involvement" OR AB "patient and public involvement") OR (TI "involving patients" OR AB "involving patients") OR (TI "caregivers involvement" OR AB "caregivers involvement") OR (TI "patient engagement" OR AB "patient engagement") OR (TI "engaging patients" OR AB "engaging patients") OR (TI "engage patients" OR AB "engage patients") OR (TI "patient participation" OR AB "patient participation") OR (MH "consumer participation+”) OR (TI "patient engagement" OR AB "patient engagement") OR (TI "patient advocacy" OR AB "patient advocacy") OR (TI "patient advocate" OR AB "patient advocate") OR (TI "patient representatives" OR AB "patient representatives") OR (TI "patient empowerment" OR AB "patient empowerment") OR (TI "health activism" OR AB "health activism") OR (TI "health advocacy" OR AB "health advocacy") OR (TI "patient input" OR AB "patient input") OR (TI "patient activation" OR AB "patient activation"))

AND

((AB(Search*) AND (AB(medline) OR AB(pubmed) OR AB(cinahl) OR AB(scopus) OR AB(embase) OR AB(psycinfo))) OR TI(“meta-analys*”) OR AB(“meta-analys*”) OR TI("meta analy*") OR AB(“meta analys*”) OR TI(metanaly*) OR AB(metanaly*) OR TI(metaanaly*) OR AB(metaanaly*) OR MH("meta analysis") OR PT("meta analysis") OR PT("systematic review") OR MH("systematic review”) OR (TI "systematic review*" OR AB "systematic review*") OR TI("systematic review") OR TI("systematic literature review") OR TI("systematic scoping review") OR TI("systematic narrative review") OR TI("systematic qualitative review") OR TI("systematic evidence review") OR TI("systematic quantitative review") OR TI("systematic meta-review") OR TI("systematic critical review") OR TI("systematic mixed studies review") OR TI("systematic mapping review") OR TI("systematic cochrane review") OR TI("systematic search and review”) OR TI("systematic integrative review"))

Box 3 : Search strategy for PsycInfo

((AB(Search*) AND (AB(medline) OR AB(pubmed) OR AB(cinahl) OR AB(scopus) OR AB(embase) OR AB(psycinfo))) OR (ME(“meta analysis”) OR TIAB(“meta analysis”) OR TIAB(("meta analyses" OR "meta analysis" OR "meta analytic")) OR TIAB(metanaly*) OR TIAB(metaanaly*) OR TIAB("met analy*") OR ME("systematic review") OR TIAB(("systematic reviews")) OR TI("systematic literature review") OR TI("systematic scoping review") OR TI("systematic narrative review") OR TI("systematic qualitative review") OR TI("systematic evidence review") OR TI("systematic quantitative review") OR

TI("systematic meta-review") OR TI("systematic critical review") OR TI("systematic mixed studies review") OR TI("systematic mapping review") OR TI("systematic cochrane review") OR TI("systematic search and review”) OR TI("systematic integrative review"))) AND (TIAB("public involvement") OR TIAB("family involvement") OR TIAB("public engagement") OR TIAB("engaging stakeholders") OR TIAB("shared decision making") OR TIAB("community participation") OR TIAB("stakeholder participation") OR MAINSUBJECT.EXACT("Client Participation") OR TIAB(“client participation”) OR TIAB("stakeholder participation") OR TIAB("community involvement") OR TIAB("consumer engagement") OR TIAB("consumer participation") OR TIAB("consumer involvement") OR TIAB("lay participation") OR TIAB("lay engagement") OR TIAB("lay involvement") OR TIAB("public and patient involvement") OR TIAB("patient involvement") OR TIAB("patient and public involvement") OR TIAB("involving patients") OR TIAB("caregivers involvement") OR TIAB("patient engagement") OR TIAB("engaging patients") OR TIAB("engage patients") OR TIAB("patient participation") OR TIAB("patient engagement") OR TIAB("patient advocacy") OR TIAB("patient advocate") OR TIAB("patient representatives") OR TIAB("patient empowerment") OR TIAB("health activism") OR TIAB("health advocacy") OR TIAB("patient input") OR TIAB("patient activation"))

Eligibility criteria

Criterion	Inclusion	Exclusion
**Publication type**	Systematic reviews published in peer-reviewed journals.	Other types of articles (e.g., review article) or reviews (e.g., scoping, realist, rapid, umbrella review). Reviews published in non-peer reviewed journals.
**Study type**	Systematic reviews of empirical studies.	Systematic reviews of studies of a descriptive type (e.g., patient experiences, concept analysis) or including gray literature.
**Language**	English.	All other languages.
**Date**	Reviews published from 2001 to 2022.	Reviews published before 2001.
**Phenomenon of Interest**	Patient and public involvement (PPI) in health care with the intention to improve practices pertaining to certain health condition(s) (e.g., care routines, organizational structure, policies) and how to achieve or evaluate PPI. The focus here is on how patients and the public can be change-agents not only in their own, but also in other patients' care.	Systematic reviews of studies<br>- not explicitly reporting on the involvement of patients and the public in health care;<br>- focused on patient engagement solely in their individual health care decisions or on how patients experience living with a certain condition;<br>- about PPI in social care,<br>- about PPI in medical and health research, in health technology assessment (HTA),<br>- about the engagement of participants as research subjects (e.g. clinical, trials)

Data extraction

Data extraction form	
	**Extracted information**
**Publication details**	Authors, year of publication, journal, doi, counties, number of studies included, review objective, study design, PPI in the review, funding, years, and language covered
**Population**	Characteristics of the participant such as age, sex, healthcare need
**Interests**	Conceptualisations: Frameworks, terms and definitions of PPI used by the reviewers;<br>Interventions, tools, techniques or methods used for implementing and/or evaluating PPI;<br>Level/degree/extent of involvement (e.g., consultation, partnership);<br>Perspective of patients/members of the public involved;<br>Reported outcome(s) of PPI or outcomes used to evaluate the effectiveness of the intervention;<br>Knowledge/research gaps
**Context**	Decision-making level (macro, meso, micro);<br>Healthcare setting (e.g., primary care, hospital care, public health, mental health, education of healthcare staff)
