# Supplementary material for: Patient and public involvement in healthcare: a systematic mapping review of systematic reviews – identification of current research and possible directions for future research
Source: BMJ Open. 2024 Sep 19;14(9):e083215. doi: 10.1136/bmjopen-2023-083215 (PMC11418490; doi:10.1136/bmjopen-2023-083215)
Supplement: online supplemental file 2 [file bmjopen-14-9-s002.pdf]

Supplement 2.

List of articles excluded after full-text review and reasons for exclusion

Author(s), year	Reason for exclusion	Decision by
Oakman et al., 2021 (1)	Systematic review searching grey literature.	AW, IB & JB
Perry et al., 2013 (2)	Systematic review searching grey literature.	AW, IB & JB
Van Deveter et al., 2015 (3)	Systematic review searching grey literature.	AW, IB & JB
Baines et al., 2019 (4)	Systematic review searching grey literature.	AW, IB & JB
James et al., 2020 (5)	Systematic review searching grey literature.	AW, IB & JB
Oldfield et al., 2019 (6)	Systematic review searching grey literature.	AW, IB & JB
Decroo et al., 2013 (7)	Systematic review searching grey literature.	AW, IB & JB
Cyril et al., 2015 (8)	Systematic review searching grey literature.	AW, IB & JB
Desai et al., 2020 (9)	Systematic review searching grey literature.	AW, IB & JB
McMullen et al., 2020 (10)	Systematic review searching grey literature.	AW, IB & JB
Snijder et al., 2015 (11)	Systematic review searching grey literature.	AW, IB & JB
Lopez-Carmen et al., 2019 (12)	Systematic review searching grey literature.	AW, IB & JB
Semrau et al., 2016 (13)	Systematic review searching grey literature.	AW, IB & JB
Boivin et al., 2018 (14)	Systematic review searching grey literature.	AW, IB & JB
Dukhanin et al., 2018 (15)	Systematic review searching grey literature.	AW, IB & JB
Kislov et al., 2018 (16)	Systematic review searching grey literature.	AW, IB & JB
Kusuwo et al., 2017 (17)	Systematic review searching grey literature.	AW, IB & JB
Fønhus et al., 2018 (18)	Systematic review searching grey literature.	AW, IB & JB
Nilsen et al., 2006 (19)	Systematic review searching grey literature.	AW, IB & JB
D'Eer et al., 2022 (20)	Systematic review searching grey literature.	IB & JB
Wiles et al., 2022 (21)	Systematic review searching grey literature.	IB & JB
Hanlon et al., 2022 (22)	Systematic review searching grey literature.	IB & JB
Canuto et al., 2022 (23)	Systematic review searching grey literature.	IB & JB
Baines et al., 2022 (24)	Systematic review searching grey literature.	IB & JB
Tobiano et al., 2022 (25)	Systematic review searching grey literature.	IB & JB
Bennett-Weston et al., 2022 (26)	Systematic review searching grey literature.	IB & JB
Viksveen et al., 2022 (27)	Systematic review searching grey literature.	IB & JB
Selva et al., 2017 (28)	Systematic review searching grey literature.	AW, IB & JB
Langton et al., 2003 (29)	Systematic review including grey literature.	AW, IB & JB
Crawford et al., 2002 (30)	Systematic review including grey literature.	AW, IB & JB
Kötter et al., 2013 (31)	Systematic review including grey literature.	AW, IB & JB
Lowe et al., 2021 (32)	Systematic review including grey literature.	AW, IB & JB
Mockford et al., 2012 (33)	Systematic review including grey literature.	AW, IB & JB
Baxter et al., 2017 (34)	Systematic review including grey literature.	AW, IB & JB
McCoy et al., 2012 (35)	Systematic review including grey literature.	AW, IB & JB
Hubbard et al., 2007 (36)	Systematic review including grey literature.	AW, IB & JB
Scholz et al., 2017 (37)	Other type of article, including reviews.	JB & IB
Sharma et al., 2017 (38)	Other type of article, including reviews.	IB & JB
Biddle et al., 2021 (39)	Other type of article, including reviews.	JB & IB
Baines and Regan de Bere, 2018 (40)	Other type of article, including systematic reviews and grey literature.	JB & VC
Tempfer et al., 2011 (41)	Other type of article, including systematic reviews.	JB & IB

Avard et al., 2010 (42)	Not a systematic review of empirical studies but of policy documents /guidelines.	JB & IB
Atkinson et al., 2011 (43)	Not a systematic review of empirical studies, mostly case reports.	IB & JB
Towle et al., 2010 (44)	Not a systematic review.	VC & JB
Wykurz and Kelly, 2002 (45)	Not a systematic review.	VC & JB
George et al., 2020 (46)	Systematic review of studies of a descriptive type (patient experiences).	JB & VC
Vaisson et al., 2021 (47)	Systematic review of studies of a descriptive type.	JB & IB
Serrano-Aguilar et al., 2015 (48)	Focus on how patients experience living with a certain condition.	JB & IB
Angel and Frederiksen, 2015 (49)	Focus on patient engagement solely in their individual health care decision.	VC & JB
Cornelissen et al., 2020 (50)	Focus on patient engagement solely in their individual health care decision.	VC & JB
McIntyre and Craig, 2015 (51)	Focus on patient engagement solely in their individual health care decision.	VC & JB
Quiñones et al., 2014 (52)	Focus on patient engagement solely in their individual health care decision.	JB & IB
Sawesi et al., 2016 (53)	Focus on patient engagement solely in their individual health care decision.	JB & IB
Ree et al., 2019 (54)	Focus on patient engagement solely in their individual health care decisions or on how patients experience living with a certain condition.	JB & VC
Kostalova et al., 2021 (55)	Focus on patient engagement solely in their individual health care decisions or on how patients experience living with a certain condition.	JB & VC
Ocrainek et al. 2017 (56)	Focus on patient engagement solely in their individual health care decisions.	JB & IB
Ziegler et al., 2022 (57)	Not explicitly reporting on the involvement of patients and the public in health care (peer-groups outside hc system!?)	IB & JB
Gysels et al., 2007 (58)	Not explicitly reporting on the involvement of patients and the public in health care and focus on patient engagement solely in their individual health care decisions.	VC & JB
Reynolds et al., 2020 (59)	Not explicitly reporting on the involvement of patients and the public in health care.	VC & JB
Suijkerbuijk et al., 2019 (60)	Not explicitly reporting on the involvement of patients and the public in health care.	JB & IB
Van Beusekom et al., 2018 (61)	Not explicitly reporting on the involvement of patients and the public in health care.	JB & IB
Joseph-Williams et al., 2014 (62)	Not explicitly reporting on the involvement of patients and the public in health care; focus on patient engagement solely in their individual health care decisions or on how patients experience living with a certain condition.	VC & JB

Sideris et al., 2021 (63)	Not explicitly reporting on the involvement of patients and the public in health care; focus on patient engagement solely in their individual health care decisions or on how patients experience living with a certain condition.	VC & JB
Dendere et al., 2019 (64)	No focus on patient and public involvement in own and other's care.	VC & JB
Wagner et al., 2019 (65)	No focus on patient/public involvement.	VC & JB
Allanson et al., 2017 (66)	No focus on patient/public involvement. Not explicitly reporting on the involvement of patients and the public in health care.	JB & IB
Martin et al., 2022 (67)	About PPI in research	IB & JB
Salimi et al., 2012(68)	About PPI in research.	JB, VC & IB
Santoro Lamelas, 2020 (69)	About PPI in research.	JB, VC & IB
Jha et al., 2010 (70)	No demarcation between real patients and simulated patients/actors.	JB & IB
Porter et al., 2019 (71)	No demarcation between real patients and simulated patients/actors.	VC & JB

References

1. Oakman J, Cahill LS, Clune S, Neilson C, Shields N, Tse T, et al. Effectiveness of health consumer representative involvement in implementation of interventions to change health professional behaviour. Int J Qual Health Care. 2021;33(1).
2. Perry J, Watkins M, Gilbert A, Rawlinson J. A systematic review of the evidence on service user involvement in interpersonal skills training of mental health students. J Psychiatr Ment Health Nurs. 2013;20(6):525-40.
3. van Deventer C, McInerney P, Cooke R. Patients' involvement in improvement initiatives: a qualitative systematic review. JBI Database System Rev Implement Rep. 2015;13(10):232-90.
4. Baines R, Donovan J, Regan de Bere S, Archer J, Jones R. Patient and public involvement in the design, administration and evaluation of patient feedback tools, an example in psychiatry: a systematic review and critical interpretative synthesis. J Health Serv Res Policy. 2019;24(2):130-42.
5. James K, Brooks H, Susanti H, Waddingham J, Irmansyah I, Keliat BA, et al. Implementing civic engagement within mental health services in South East Asia: a systematic review and realist synthesis of current evidence. Int J Ment Health Syst. 2020;14(0):17.
6. Oldfield BJ, Harrison MA, Genao I, Greene AT, Pappas ME, Glover JG, et al. Patient, Family, and Community Advisory Councils in Health Care and Research: a Systematic Review. J Gen Intern Med. 2019;34(7):1292-303.
7. Decroo T, Rasschaert F, Telfer B, Remartinez D, Laga M, Ford N. Community-based antiretroviral therapy programs can overcome barriers to retention of patients and decongest health services in sub-Saharan Africa: a systematic review. Int Health. 2013;5(3):169-79.
8. Cyril S, Smith BJ, Possamai-Inesedy A, Renzaho AM. Exploring the role of community engagement in improving the health of disadvantaged populations: a systematic review. Glob Health Action. 2015;8:29842.

9. Desai S, Misra M, Das A, Singh RJ, Sehgal M, Gram L, et al. Community interventions with women's groups to improve women's and children's health in India: a mixed-methods systematic review of effects, enablers and barriers. BMJ Glob Health. 2020;5(12).
10. McMullen JMG, M.; Ingman, B. C.; Pulling Kuhn, A.; Graham, D. J.; Carson, R. L. A Systematic Review of Community Engagement Outcomes Research in School-Based Health Interventions. J Sch Health. 2020;90(12):985-94.
11. Snijder M, Shakeshaft A, Wagemakers A, Stephens A, Calabria B. A systematic review of studies evaluating Australian indigenous community development projects: the extent of community participation, their methodological quality and their outcomes. BMC Public Health. 2015;15:1154.
12. Lopez-Carmen V, McCalman J, Benveniste T, Askew D, Spurling G, Langham E, et al. Working together to improve the mental health of indigenous children: A systematic review. Children & Youth Services Review. 2019;104:104408-.
13. Semrau M, Lempp H, Keynejad R, Evans-Lacko S, Mugisha J, Raja S, et al. Service user and caregiver involvement in mental health system strengthening in low- and middle-income countries: systematic review. BMC Health Serv Res. 2016;16:79.
14. Boivin A, L'Espérance A, Gauvin FP, Dumez V, Macaulay AC, Lehoux P, et al. Patient and public engagement in research and health system decision making: A systematic review of evaluation tools. Health Expect. 2018;21(6):1075-84.
15. Dukhanin V, Topazian R, DeCamp M. Metrics and Evaluation Tools for Patient Engagement in Healthcare Organization- and System-Level Decision-Making: A Systematic Review. Int J Health Policy Manag. 2018;7(10):889-903.
16. Kislov R. WPM, Knowles S., Boaden R. Learning from the emergence of NIHR Collaborations for Leadership in Applied Health Research and Care (CLAHRCs): a systematic review of evaluations. 2018. 2018;13:111-27.
17. Kusuwo P, Myezwa H, Pilusa S, M'Kumbuzi V. A systematic review to identify system-related elements that can be used to evaluate community-based rehabilitation (CBR) programmes. European Journal of Physiotherapy. 2017;19:41-6.
18. Fønhus MS, Dalsbø TK, Johansen M, Fretheim A, Skirbekk H, Flottorp SA. Patient-mediated interventions to improve professional practice. Cochrane Database Syst Rev. 2018;9(9):Cd012472.
19. Nilsen ES, Myrhaug HT, Johansen M, Oliver S, Oxman AD. Methods of consumer involvement in developing healthcare policy and research, clinical practice guidelines and patient information material. Cochrane Database Syst Rev. 2006;2006(3):Cd004563.
20. D'Eer L, Quintiens B, Van den Block L, Dury S, Deliens L, Chambaere K, et al. Civic engagement in serious illness, death, and loss: A systematic mixed-methods review. Palliative Medicine. 2022;36(4):625-51.
21. Wiles LK, Kay D, Luker JA, Worley A, Austin J, Ball A, et al. Consumer engagement in health care policy, research and services: A systematic review and meta-analysis of methods and effects. PLoS One. 2022;17(1):e0261808.
22. Hanlon CA, McIlroy D, Poole H, Chopra J, Saini P. Evaluating the role and effectiveness of co-produced community-based mental health interventions that aim to reduce suicide among adults: A systematic review. Health Expectations. 2023;26(1):64-86.
23. Canuto K, Preston R, Rannard S, Felton-Busch C, Geia L, Yeomans L, et al. How and why do women's groups (WGs) improve the quality of maternal and child health (MCH) care? A systematic review of the literature. BMJ Open. 2022;12(2):e055756.
24. Baines R, Bradwell H, Edwards K, Stevens S, Prime S, Tredinnick-Rowe J, et al. Meaningful patient and public involvement in digital health innovation, implementation and evaluation: A systematic review. Health Expectations. 2022;25(4):1232-45.
25. Tobiano G, Roberts S, Muir R, Jerofke-Owen T, Ting C, Thorning S, et al. Patient-mediated interventions in hospital: A systematic review. Journal of Advanced Nursing. 2023;79(2):418-41.

26. Bennett-Weston A, Gay S, Anderson ES. A theoretical systematic review of patient involvement in health and social care education. Advances in Health Sciences Education. 2023;28(1):279-304.
27. Viksveen P, Bjønness SE, Cardenas NE, Game JR, Berg SH, Salamonsen A, et al. User involvement in adolescents' mental healthcare: a systematic review. European Child & Adolescent Psychiatry. 2022;31(11):1765-88.
28. Selva AS, A. J.; Pequeño, S.; Zhang, Y.; Solà, I.; Pardo-Hernandez, H.; Selva, C.; Schünemann, H.; Alonso-Coello, P. Incorporating patients' views in guideline development: a systematic review of guidance documents. J Clin Epidemiol. 2017;88:102-12.
29. Langton H, Barnes M, Haslehurst S, Rimmer J, Turton P. Collaboration, user involvement and education: a systematic review of the literature and report of an educational initiative. Eur J Oncol Nurs. 2003;7(4):242-52.
30. Crawford MJ, Rutter D, Manley C, Weaver T, Bhui K, Fulop N, et al. Systematic review of involving patients in the planning and development of health care. Bmj. 2002;325(7375):1263.
31. Kötter T, Schaefer FA, Scherer M, Blozik E. Involving patients in quality indicator development - a systematic review. Patient Prefer Adherence. 2013;7:259-68.
32. Lowe D, R. R, L. S, B. M, L. W, L. G-W, et al. Effects of consumers and health providers working in partnership on health services planning, delivery and evaluation. Cochrane Database of Systematic Reviews. 2021(9).
33. Mockford C, Staniszewska S, Griffiths F, Herron-Marx S. The impact of patient and public involvement on UK NHS health care: a systematic review. Int J Qual Health Care. 2012;24(1):28-38.
34. Baxter S, Clowes M, Muir D, Baird W, Broadway-Parkinson A, Bennett C. Supporting public involvement in interview and other panels: a systematic review. Health Expect. 2017;20(5):807-17.
35. McCoy DC, Hall JA, Ridge M. A systematic review of the literature for evidence on health facility committees in low- and middle-income countries. Health Policy Plan. 2012;27(6):449-66.
36. Hubbard GK, L.; Donaghy, E.; McDonald, C.; Kearney, N.; Hubbard, Gill; Kidd, Lisa; Donaghy, Edward; McDonald, Charlotte; Kearney, Nora. A review of literature about involving people affected by cancer in research, policy and planning and practice. Patient Education & Counseling. 2007;65(1):21-33.
37. Scholz B, Gordon S, Happell B. Consumers in mental health service leadership: A systematic review. Int J Ment Health Nurs. 2017;26(1):20-31.
38. Sharma AE, Knox M, Mleczko VL, Olayiwola JN. The impact of patient advisors on healthcare outcomes: a systematic review. BMC Health Serv Res. 2017;17(1):693.
39. Biddle MSY, Gibson A, Evans D. Attitudes and approaches to patient and public involvement across Europe: A systematic review. Health Soc Care Community. 2021;29(1):18-27.
40. Baines RL, Regan de Bere S. Optimizing patient and public involvement (PPI): Identifying its "essential" and "desirable" principles using a systematic review and modified Delphi methodology. Health Expect. 2018;21(1):327-35.
41. Tempfer CB, Nowak P. Consumer participation and organizational development in health care: a systematic review. Wien Klin Wochenschr. 2011;123(13):408-14.
42. Avard D, Jean MS, Grégoire G, Page M. Public involvement in health genomics: The reality behind the policies. International Journal of Consumer Studies. 2010;34(5):508-24.
43. Atkinson JA, Vallely A, Fitzgerald L, Whittaker M, Tanner M. The architecture and effect of participation: a systematic review of community participation for communicable disease control and elimination. Implications for malaria elimination. Malar J. 2011;10:225.
44. Towle A, Bainbridge L, Godolphin W, Katz A, Kline C, Lown B, et al. Active patient involvement in the education of health professionals. Medical Education. 2010;44(1):64-74.
45. Wykurz G, Kelly D. Developing the role of patients as teachers: Literature review. BMJ: British Medical Journal. 2002;325(7368):818-21.

46. George S, Zacher Dixon L, Carrasco E, Romo O, Vides L, Balcazar H. Talk to PAPA: A Systematic Review of Patient/Participant (PAPA) Feedback on the Interactions with Community Health Workers using a Depth Analysis Approach. Journal of Ambulatory Care Management. 2020;43(1):55-70.
47. Vaisson G, Provencher T, Dugas M, Trottier M, Chipenda Dansokho S, Colquhoun H, et al. User Involvement in the Design and Development of Patient Decision Aids and Other Personal Health Tools: A Systematic Review. Med Decis Making. 2021;41(3):261-74.
48. Serrano-Aguilar PT-MM, M.; Pérez de la Rosa, A.; Cuellar-Pompa, L.; Saavedra-Medina, H.; Linertova, R.; Perestelo-Perez, L.; Perez-Ramos, J.; Rivero-Santana, A. Patient participation in a Clinical Guideline Development for Systemic Lupus Erythematosus. Patient Educ Couns. 2015;98(9):1156-63.
49. Angel S, Frederiksen KN. Challenges in achieving patient participation: A review of how patient participation is addressed in empirical studies. Int J Nurs Stud. 2015;52(9):1525-38.
50. Cornelissen D, de Kunder S, Si L, Reginster JY, Evers S, Boonen A, et al. Interventions to improve adherence to anti-osteoporosis medications: an updated systematic review. Osteoporos Int. 2020;31(9):1645-69.
51. McIntyre R, Craig A. A Literature Review of Patient Education: Is IT Time to Move Forward? J Med Imaging Radiat Sci. 2015;46(3):S75-s85.
52. Quiñones AR, Richardson J, Freeman M, Fu R, O'Neil ME, Motu'apuaka M, et al. Educational group visits for the management of chronic health conditions: a systematic review. Patient Educ Couns. 2014;95(1):3-29.
53. Sawesi S, Rashrash M, Phalakornkule K, Carpenter JS, Jones JF. The Impact of Information Technology on Patient Engagement and Health Behavior Change: A Systematic Review of the Literature. JMIR Med Inform. 2016;4(1):e1.
54. Ree E, Wiig S, Manser T, Storm M. How is patient involvement measured in patient centeredness scales for health professionals? A systematic review of their measurement properties and content. BMC Health Serv Res. 2019;19(1):12.
55. Kostalova BR, J.; Dobbels, F.; Gerull, S.; Mala-Ladova, K.; Zullig, L. L.; De Geest, S. Medication adherence interventions in transplantation lack information on how to implement findings from randomized controlled trials in real-world settings: A systematic review. Transplant Rev (Orlando). 2021;36(1):100671.
56. Okrainec K, Lau D, Abrams HB, Hahn-Goldberg S, Brahmbhatt R, Huynh T, et al. Impact of patient-centered discharge tools: A systematic review. J Hosp Med. 2017;12(2):110-7.
57. Ziegler E, Hill J, Lieske B, Klein J, dem Knesebeck Ov, Kofahl C. Empowerment in cancer patients: Does peer support make a difference? A systematic review. Psycho-Oncology. 2022;31(5):683-704.
58. Gysels M, Richardson A, Higginson IJ. Does the patient-held record improve continuity and related outcomes in cancer care: a systematic review. Health Expect. 2007;10(1):75-91.
59. Reynolds TL, Ali N, Zheng K. What Do Patients and Caregivers Want? A Systematic Review of User Suggestions to Improve Patient Portals. AMIA Annu Symp Proc. 2020;2020:1070-9.
60. Suijkerbuijk S, Nap HH, Cornelisse L, WA IJ, de Kort YAW, Minkman MMN. Active Involvement of People with Dementia: A Systematic Review of Studies Developing Supportive Technologies. J Alzheimers Dis. 2019;69(4):1041-65.
61. van Beusekom MM, Kerkhoven AH, Bos MJW, Guchelaar HJ, van den Broek JM. The extent and effects of patient involvement in pictogram design for written drug information: a short systematic review. Drug Discov Today. 2018;23(6):1312-8.
62. Joseph-Williams NE, G.; Edwards, A. Knowledge is not power for patients: a systematic review and thematic synthesis of patient-reported barriers and facilitators to shared decision making. Patient Educ Couns. 2014;94(3):291-309.
63. Sideris GA, Vyllioti AT, Dima D, Chill M, Njuguna N. The Value of Web-Based Patient Education Materials on Transarterial Chemoembolization: Systematic Review. JMIR Cancer. 2021;7(2):e25357.

64. Dendere R, Slade C, Burton-Jones A, Sullivan C, Staib A, Janda M. Patient Portals Facilitating Engagement With Inpatient Electronic Medical Records: A Systematic Review. J Med Internet Res. 2019;21(4):e12779.
65. Wagner A, Radionova N, Rieger MA, Siegel A. Patient Education and Continuing Medical Education to Promote Shared Decision-Making. A Systematic Literature Review. Int J Environ Res Public Health. 2019;16(14).
66. Allanson ER, Tunçalp Ö, Vogel JP, Khan DN, Oladapo OT, Long Q, et al. Implementation of effective practices in health facilities: a systematic review of cluster randomised trials. BMJ Glob Health. 2017;2(2):e000266.
67. Martin S, Chamberlain C, Rivett A, Selman LE. How are public engagement health festivals evaluated? A systematic review with narrative synthesis. PLoS One. 2022;17(8):e0267158.
68. Salimi Y, Shahandeh K, Malekafzali H, Loori N, Kheiltash A, Jamshidi E, et al. Is Community-based Participatory Research (CBPR) Useful? A Systematic Review on Papers in a Decade. Int J Prev Med. 2012;3(6):386-93.
69. Santoro Lamelas V. The practice of action research on health in Latin America: A systematic review. Educational Action Research. 2020;28(2):293-309.
70. Jha V, Setna Z, Al-Hity A, Quinton ND, Roberts TE. Patient involvement in teaching and assessing intimate examination skills: a systematic review. Med Educ. 2010;44(4):347-57.
71. Porter J, Kellow N, Anderson A, Bryce A, Dart J, Palermo C, et al. Patient Involvement in Education of Nutrition and Dietetics Students: A Systematic Review. Nutrients. 2019;11(11).
