# Supplementary material for: Patient and public involvement in healthcare: a systematic mapping review of systematic reviews – identification of current research and possible directions for future research
Source: BMJ Open. 2024 Sep 19;14(9):e083215. doi: 10.1136/bmjopen-2023-083215 (PMC11418490; doi:10.1136/bmjopen-2023-083215)
Supplement: online supplemental file 3 [file bmjopen-14-9-s003.pdf]

## *Supplement 3.*

**Table 1** **Healthcare quality improvement**

| Authors | Interest | PPI in review process | Aim | Review design | Relevant findings | Research gaps identified | Desired outcomes of primary studies |
|---|---|---|---|---|---|---|---|
| Bombard et al. 2018 | **Design, delivery, and evaluation of health services** | NR | Identify strategies and contextual factors that enable optimal patient engagement in design, delivery, and evaluation of health services | Thematic analysis of 48 studies: qualitative (27), quantitative (3), mixed methods (13), other (5) | The level of engagement appears to influence the outcomes of service redesign. Strategies and contextual factors identified were related to techniques to enhance design, recruitment, involvement, and leadership, as well as creating a receptive context. | Patients' experiences of the engagement process,<br>whether outcomes translate into improved quality of care, "<br>conceptual muddle" is one of the greatest barriers to truly integrating patient involvement into health services, policy, and research | Discrete products, care processes, care service delivery, governance |
| Danhoundo et al. 2018 | **Improving social accountability processes in the health sector** | NR | Identify the conditions that facilitate effective social accountability in sub-Saharan Africa | Narrative synthesis of 14 studies: qualitative (7), mixed method (5), quantitative (2) | Effective social accountability interventions involve leveraging partnerships and building coalitions; being context-appropriate; integrating data and information collection and analysis; clearly defined roles, standards, and responsibilities of leaders; and meaningful citizen engagement. Health system barriers, corruption, fear of reprisal, and limited funding appear to be major challenges to effective social accountability interventions. The variability of outcome measures and reporting standards make it difficult to comment on overall effects. | Future studies implementing social accountability interventions should include sufficiently long periods of follow-up to determine the sustainability of such programs. | Health service quality, health indicators |
| Evans et al. 2010 | **Impact of participatory approaches by UK public health units on health and social outcomes** | NR | Review the impact of participatory approaches by UK<br>NHS public health units | Narrative analysis of 7 studies: qualitative (4), mixed method (3) | There is a gap between rhetoric and reality. Studies were poor in quality. Very little evidence exists of participatory approaches by UK public health units having any noteworthy impact on health or social outcomes. | Need for high quality evaluations to assess the quality and effectiveness of participatory approaches within UK NHS public health | Health outcomes, process and experience of participation, community awareness |

| | | | | | | | |
|---|---|---|---|---|---|---|---|
| Green et al. 2020 | **Experience-based co-design (EBCD) in health service improvement** | NR | Examine the use and reporting of EBCD in health service improvement | Narrative synthesis of 20 studies. | EBCD is used predominantly for quality improvement but has potential to be used for intervention design projects. There is variation in the use of EBCD, with many studies eliminating or modifying some EBCD stages. Moreover, there is no consistency in reporting. | A reporting guideline needs to be established to encourage researchers to conduct and report EBCD projects in a consistent manner to enable replication. | Service or care pathways, process outcomes |
| Haldane et al. 2019 | **Community participation in health service development, implementation, and evaluation** | NR | Examine outcomes of community involvement in health service planning, implementation, monitoring, and evaluation for a variety of diseases | Narrative synthesis of 49 studies:<br>quantitative (22), qualitative (14), case studies (13) | Community participation yields positive outcomes on organizational, community and individual level, particularly when substantiated by strong organisational and community processes. | More robust program evaluations, measurement of long-term outcomes and cost-effectiveness in more settings globally | Process outcomes, community outcomes, health outcomes, empowerment, stakeholder perspectives |
| Kesale et al. 2022 | **Health facility governing committees (HFGC)** | NR | Review the effects of decentralization on the functionality of HFGCs in low- and middle-income countries | Narrative synthesis of 24 studies: qualitative (14), quantitative (10) | The study has found many HFGCs to have very low functionality, while a few HFGCs are performing very well. Limited functionality is due to unawareness of the scope of their responsibilities and powers, insufficient resources, lack of support from higher levels, and committee composition. | Committees should be capacitated in their tasks and powers and given full autonomy in order to realize their full potential. | Governance outcomes |
| Lloyd et al. 2021 | **Health-service outcomes of public involvement in health service design** | NR | Identify and synthesize health service outcomes of public involvement and document how outcomes were evaluated | Narrative synthesis of 93 studies: qualitative (55), mixed methods (34), quantitative (4) | A range of health service outcomes were reported at various levels (service level, across services, and across organisations). Evaluations of outcomes were reported in less than half of the studies. There are challenges due to inadequate descriptions of study design and poor reporting. | Urgent need for evaluations, including longitudinal study designs and cost-benefit analyses | Discrete products, improvements to health services, system or policy levels change |
| Moore et al. 2019 | **Participatory methods in eHealth resource development** | Yes | Summarize the evidence about participatory methods and frameworks used to engage health service users (HSU) in the development of eHealth resources | Quantitative synthesis of 90 studies and qualitative synthesis of 30 studies (after quality assessment) | 24 participatory frameworks or approaches were identified; however, many studies didn't reference a framework. Effective involvement of health service users resulted in improved relevance of the eHealth resource, improved responsiveness and ownership. | Evaluation of participatory methodology was reported inconsistently. A standardized tool to evaluate processes and outcomes from the HSU perspective, may bring more objectivity to evaluation. | Discrete products |

| Table 2 Patient safety | | | | | | | |
|---|---|---|---|---|---|---|---|
| **Authors** | **Interest** | **PPI in review process** | **Aim** | **Review design** | **Relevant findings** | **Research gaps identified** | **Desired outcomes of primary studies** |
| Giap and Park 2021 | **Patient and family involvement interventions for promoting patient safety**<br><br>Patients in various disease situations from acute to chronic, aged from children to adults, and ranged from medical to surgical patients who received primary or secondary healthcare, as well as their family caregivers. | NR | Evaluate and quantify the effects of patient and family involvement (PFI) interventions on patient safety | Meta-analysis of 22 studies: RCT (18) and non-RCT (4) | PFI were beneficial in significantly reducing adverse events, decreasing the length of hospital stay, increasing patient safety experiences, and improving patient satisfaction. PFI interventions did not significantly enhance the perception of patient safety or the quality of life. Moderate-to-high heterogeneity was found for all impacts except adverse events and length of hospital stay. | Lack of consensus about the definition of PFI and its components, type of PFI interventions and patient safety outcomes, steps and methods to implement PFI<br>. | Adverse events,<br>patient safety experiences, perception of patient safety, length of hospital stay,<br>patient satisfaction,<br>QoL |
| Lee et al. 2021 | **Patient and family involvement for patient safety** | NR | Identify the types of interventions to improve patient safety that focused on engaging patients and their families, and the effectiveness of these interventions | Narrative synthesis of 15 studies: quasi-experimental (11), RCT (4) | The studies used intervention strategies at the "information" and "involvement" engagement levels. Interventions with strategies only at the information level mostly measured safety perception and were mostly found to be effective. Interventions with both information and involvement strategies measured more diverse outcomes, but their effectiveness was inconsistent. | More rigorous methodologies needed | Safety-related outcomes:<br>Patient safety events,<br>clinical outcomes, safety perception,<br>self-efficacy,<br>safety knowledge, collaboration,<br>decision-making, safety behavior |
| Park and Giap 2020 | **Impact of Patient and Family Engagement interventions on patient safety** | NR | Provide a comprehensive insight into the impact of patient and family engagement interventions on patient safety, the related issues and the perspectives of the people involved in implementing this approach | Narrative synthesis of 42 studies: RCT (8), NRS (9), qualitative surveys (12), qualitative studies (13) | Common intervention groups included 'direct care' and 'organization' levels with 'consultation' and 'involvement' approaches, while the 'health system' level and 'partnership and shared leadership' approaches were rarely implemented. Findings revealed positive effects of the interventions on patient safety. | Consensus guidelines for implementing patient and family engagement in patient safety | Perception and awareness of risks,<br>adverse events,<br>patient and family satisfaction,<br>health outcomes,<br>length of stay,<br>attitudes,<br>safety climate,<br>attitudes of HCPs,<br>work environment of HCPs |

| Table 3 Community-based initiatives | | | | | | | |
|---|---|---|---|---|---|---|---|
| **Authors** | **Interest** | **PPI in review** | **Aim** | **Review design** | **Relevant findings** | **Research gaps identified** | **Desired outcomes of primary studies** |
| Banna and Bersamin 2018 | **Community involvement in nutrition interventions for Indigenous populations in the US** | NR | Identify and characterize nutrition interventions conducted with Indigenous populations in the US, and to determine whether and to what degree communities are involved in intervention design, implementation and evaluation | Narrative synthesis of 49 studies: RCT (11), pre-post studies (24), pre-post studies embedded in RCT (5), other studies (9) | Involvement of communities in intervention design, implementation, and evaluation varied from not at all to involvement at all stages. Of programs reporting significant changes in outcomes, more than half used at least three strategies to engage communities. However, formative research to inform the evaluation was not performed to a great degree. | Studies focused on Indigenous youth | Diet/food behaviours, physical activity, weight status, knowledge/awareness/self-efficacy, other health outcomes |
| Farnsworth et al. 2014 | **Community participation for enhancement in child survival and early development**<br><br>Low- and middle-income countries | Yes, co-author | Assess the role of community participation in contributing to improved population-level infant and child health outcomes, mediated through improved household practices or better care-seeking behavior | Evidence review of 34 studies: RCT (17), pre-post studies (6), case-control studies (3), other studies (8) | Interventions designed to maximize community collaboration and participation can have a beneficial impact on child health. Some but limited evidence hints that the effects are potentially sustainable in some circumstances. There is some evidence that community mobilization efforts can be cost-effective. | Lack of process evaluations of child health and development research, including community engagement in self-evaluation, evidence on types of community engagement resulting in improved child outcomes, sustainability, research in countries where the challenges for child survival are greatest, in particular Sub-Saharan Africa | Health outcome indicators, knowledge, attitude and/or practice, intervention coverage, costs |
| Haldane et al. 2020 | **Chronic condition programs**<br><br>High- and upper-middle income countries, majority mental health | NR | Examine the evidence on how communities are involved in planning and implementing chronic condition programs | Narrative synthesis of 32 studies: qualitative (14), case studies (7), observational studies (5), RCT (3), mixed methods (2), non-RCT intervention studies (1) | Challenges for community involvement included user factors, other stakeholder factors, human resource factor and contextual challenges | Evaluation of health outcomes, both short and long-term, as well as cost-effectiveness | User and staff perspectives, awareness, health literacy, social oriented outcomes, health status |
| Heintze et al. 2007 | **Community-based dengue** | NR | Analyse evidence regarding the achievements of | Narrative review of 11 studies: RCT (2), controlled before | Evidence that community-based dengue control programmes alone and in combination with | Better understanding needed which specific components of the intervention strategy in | Classical entomological indices, dengue incidence rates/number of cases |

| | | | | | | | |
|---|---|---|---|---|---|---|---|
| | **control programmes**<br><br>Tropical countries | | community-based dengue control programmes | and after trials (6), interrupted time series (3) | other control activities can enhance the effectiveness of dengue control programmes is weak. Methodological weaknesses were found in all studies. | combination with community participation and/or other partnership, have the greatest impact on dengue control and are cost effective, sustainability and monitoring of strategies | |
| Hoon Chuah et al. 2018 | **Community participation for general health initiatives**<br><br>High- and upper-middle income countries | NR | Examine the evidence for community participation in relation to general, non-disease specific health initiatives, including the use of theories to inform community participation | Narrative synthesis of 79 studies: program descriptions (36), qualitative (22), quantitative (13), mixed-methods (8) | Community participation in general health initiatives can indeed contribute to positive effects in terms of service, social and health outcome. Community participation is a complex process that is strongly influenced by the context in which it occurs. | Need for studies grounded theoretically and with robust study designs | Community outcomes,<br>service outcomes,<br>health outcomes,<br>process outcomes |
| Kerrigan et al. 2013 | **Community empowerment among female sex workers preventing HIV**<br><br>Low- and middle income countries | Yes | Systematically review the peer-reviewed evidence regarding the impact of community empowerment as an HIV prevention strategy among sex workers in low- and middle-income countries | Meta-analysis of 10 studies: RCT (1), observational studies (9) | Positive effects were documented on multiple HIV-related outcomes including HIV infection, sexually transmitted infections, and consistent condom use between sex workers and their clients. | Development and application of context-specific measures of community empowerment among sex workers, evaluation of the impact on HIV incidence, also for transgender and male sex workers | HIV-infection,<br>sexually transmitted-infections,<br>condom use |
| Moore et al. 2014 | **Community empowerment and involvement of female sex workers**<br><br>Africa | NR | Systematically review community empowerment processes for female sex workers as well as sexual and reproductive health projects in Africa | Narrative review of 129 studies (42 projects) | Empowerment processes were usually limited to peer-education. Community mobilisation was rarely documented and while most projects successfully engaged communities, few progressed to involvement, community ownership or sustainability. Only a few interventions had evolved to facilitate democratic structures. These reported improved sexual negotiating power, community solidarity,positive behavioural and clinical outcomes. | Appropriate methods and follow-up periods to measure intervention outcomes longitudinally | Behavioural and clinical outcomes,<br>community empowerment,<br>process outcomes |
| Prost et al. 2013 | **Women's groups practicing** | NR | Systematically review randomized | Meta-analysis of 7 RCT studies | Women's groups practicing participatory learning and action | Comparison of the determinants of differences in | Birth outcomes,<br>neonatal and maternal mortality, |

| | | | | | | | |
|---|---|---|---|---|---|---|---|
| | **participatory learning and action to improve maternal and newborn health**<br><br>Low-resource settings, low- and middle-income countries | | controlled trials to assess the effect of women's groups practicing participatory learning and action | | led to substantial reductions in neonatal and maternal mortalities in rural, low-resource settings. The proportion of pregnant women participating in groups and the population coverage of groups were key predictors of the effect. The intervention was cost effective by WHO standards and could save an estimated 283000 newborn infants and 36600 mothers per year if implemented in rural areas of 74 Countdown countries. | costs, or the effect of scale on cost, scale up of such interventions and integration into health systems | cost-effectiveness |
| Rass et al. 2020 | **Community participation in healthcare responses to crisis**<br><br>Low- and middle-income countries | NR | Understand the role of community participation in humanitarian health responses for conflict-affected populations in low- and middle-income countries and the barriers and facilitators to community participation in healthcare responses | Descriptive thematic synthesis of 18 studies: qualitative (15), quantitative (2), mixed method (1) | Community participation can strengthen humanitarian health responses in the form of improved acceptability, awareness, access, quality, and sustainability of health services. However, included studies were of mixed quality and the overall strength of evidence was weak. There was limited critical engagement with concepts of participation. | Effects of community participation on individual and health system outcomes, need for research on more meaningful community participation, particularly in framing problems and creating solutions | Individual health outcomes, service access and utilization, community acceptability and awareness.<br>quality of services |
| Sharma et al. 2018 | **Community participation for improved maternal health**<br><br>South Asia | NR | Systematically review the effectiveness of community interventions in improving maternal healthcare outcomes in South Asia. | Meta-analysis of 11 randomized trials | Evidence is of low quality. Community interventions showed a small improvement in the number of women attending at least on antenatal care visit. Healthcare facility births were modestly increased in women's education groups. Risk of maternal deaths after 2 and 3 years were no different between women's education groups and control. | Impact of male involvement in community interventions | Attendance to antenatal care and giving birth at healthcare facilities, maternal mortality |

**Table 4** **Peer-support**

| Authors | Interest | PPI in review process | Aim | Review design | Relevant findings | Research gaps identified | Desired outcomes of primary studies |
|---|---|---|---|---|---|---|---|
| Gaiser et al. 2021 | **Peer providers in behavioral health workforce**<br><br>Mental healthcare/substance use (Veterans affairs, hospital, community health facilities) | NR | Better define the roles of peers and their unique contributions to behavioural healthcare | Evidence synthesis of 23 studies: RCT (14), quasi-experimental (3), cohort analytic (2), cross-sectional (1), retrospective comparison group study (1), retrospective survey (1), RCT secondary analysis (1) | Findings from current literature on the efficacy of peer providers are modest owing to methodologic shortcomings. Although modest, findings suggest that peer providers can be effective and important providers in preventing and treating behavioral health disorders. | Cost and expenditures associated with peer-delivered services, efficacy of peer-provided services, the degree to which they contribute to improved patient outcomes, and the circumstances and roles that are most effective | Clinical improvements,<br>social functioning,<br>QoL,<br>patient activation,<br>behavioral health |
| Genberg et al. 2016 | **Interventions involving peers in the HIV care cascade**<br><br>Sub-Saharan Africa and others | NR | Systematically review studies that used HIV-positive peers to bolster linkage, retention, and/or adherence to antiretroviral therapy (ART) | Narrative review of 9 studies: RCT (6), cluster-RCT (2), cross-sectional (1) | Despite strong quality ratings of the included studies, consistent evidence does not yet exist. Overall findings were mixed on the impact of peers on ART adherence, viral suppression, and mortality. While positive effects of peer interventions on improving linkage and retention were found, there were limited studies assessing these outcomes. | Future work in carefully designed studies with sufficient power to detect modest effects could help demonstrate the effectiveness of peers. | Adherence to medical treatment,<br>mortality,<br>viral suppression,<br>linkage and retention |
| Pitt et al. 2013 | **Consumer-providers in mental health**<br><br>Mental health services | Yes | Assess the effects of employing current or past adult consumers of mental health services as providers of statutory mental health services | Meta-analysis, RCT studies (11) | There is low to moderate quality evidence indicating that involving consumer-providers in mental health teams results in psychosocial, mental health symptom and service use outcomes for clients that are no better or worse than those achieved by professionals employed in similar roles. There is no evidence of harm associated with involving consumer-providers in mental health teams. | Future randomised controlled trials of consumer-providers in mental health service provision should minimise bias through the use of adequate randomisation and concealment of allocation, blinding of outcomes where possible, the comprehensive reporting of outcome data, and the avoidance of contamination between treatment groups. | Psychosocial outcomes,<br>mental health symptoms,<br>service use,<br>client satisfaction |
| Satinsky et al. 2021 | **Peer-delivered services for substance use in** | NR | Synthesize the evidence of peer-delivered services | Narrative review of 34 studies: qualitative studies (7), cross- | High risk of bias but some articles demonstrated positive impact of the peer-delivered services, | Need for standardized outcomes to allow for better comparison across studies, | Substance use,<br>risk-behavior,<br>wellbeing, |

| | | | | | | | |
|---|---|---|---|---|---|---|---|
| | **low- and middle-income countries** | | for substance use in in low- and middle-income countries | sectional (7), cohort studies (2), quasi-experimental (7), descriptive (3), RCT (9), mixed methods (1), sub-analysis of 10 studies with EPOC framework | including reduced risk behaviors and increased knowledge of infectious disease, while many others showed no significant difference in outcomes between peer intervention and control groups. | need for a better understanding of the peer role across contexts, opportunities for bi-directional learning and capacity building between high-, low- and middle-income countries | depression,<br>increased testing for infectious disease,<br>adherence to treatment,<br>HIV incidence |
| Simpson and House 2002 | **User-involvement in mental health services** | NR | Identify evidence from comparative studies on the effects of involving users in the delivery and evaluation of mental health services | Narrative synthesis of 12 studies: RCT (5), comparative studies (5), descriptive studies (2) | Users of mental health services can be involved as employees of such services, trainers, or researchers without damaging them. In some studies, benefit was indicated for clients of employees who were or who had been users of services, and, although this was not present across all studies, there were no serious disadvantages. | Methodologically stronger studies are needed. More evidence for effectiveness and more formal evaluations are needed. | Psychosocial outcomes (i.e., employment),<br>symptoms,<br>service use,<br>client satisfaction |
| Verma et al. 2022 | **Peer coach-led type 2 diabetes mellitus self-management** | Yes, co-author | Summarize evidence on the impact of peer coach-led type 2 diabetes mellitus self-management interventions on glycemic control and self-management outcomes | Meta-analysis of RCT studies (13) | Peer coaching improved HbA1c levels, quality of life, self-efficacy, diabetes distress and patient activation, but there is inconclusive effect on medication adherence, hypoglycemic symptoms, diabetes specific social support and depression. | Additional evidence is needed to continue unravelling what constitutes efficient peer-led self-management interventions for type 2 diabetes mellitus, understand patient preferences for interventions and how to integrate them into clinical practice and outreach sectors. | Glycemic control,<br>self-management,<br>adherence to treatment,<br>depression,<br>QoL |

**Table 5** **Education of healthcare professionals**

| Authors | Interest | PPI in review | Aim | Review design | Relevant findings | Research gaps identified | Desired outcomes of primary studies |
|---|---|---|---|---|---|---|---|
| Dijk et al. 2020 | **Patient involvement in undergraduate medical education** | Yes, co-author | Identify the scope, knowledge gaps relating to rationale and motivation for involvement, recruitment, preparation, roles, learning outcomes, key procedural contributors | Narrative synthesis of 49 studies: qualitative (38), quantitative (6), mixed methods designs (5) | Patients were engaged in a variety of educational settings in and outside of the hospital. The vast majority of studies describe patients taking on the role of a patient teacher and formative assessor. Patients are increasingly involved in course and curriculum development, student selection and summative assessment. | Financial aspects related to patient involvement. Lack of common terminology.<br>Long-term effects for patients, students and the healthcare system, especially on the subjects of patient-centredness and shared decision-making. Economic evaluation of patient involvement. Update existing frameworks for patient involvement to the newly identified roles and needs patients have in medical education | Professional competencies |
| Finch et al. 2018 | **Impact of patient feedback on healthcare students learning** | NR | Review how feedback from patients impact upon healthcare student clinical skill development and learning | Narrative synthesis of 12 studies: quantitative (7), qualitative (2), mixed methods (3) | Eleven studies reported that patient feedback improved students' clinical skills.<br><br>Patient feedback may be a useful component of student learning.<br><br>Patient feedback is a potentially powerful but underutilized technique in student training and, given its strong alignment with the principles of patient-centered care, needs to be examined further. | More studies with rigorous methodologies are required. Need for further research into the effects of patient feedback on allied health student learning and into patients with a variety of disorders, including communication disorders. Identification of the optimal way for patients to deliver feedback to healthcare students and whether the ideal feedback method varies between disciplines. Best methods to evaluate the effect of patient feedback. Lack of validated tools. | Clinical and communication skill development |
| Gordon et al. 2020 | **Patient/service user involvement in medical education** | Yes, 2 co-authors | To examine patient and service user involvement, the potential outcome of such involvement and 'why' such involvement impacts students | Narrative synthesis of 39 studies: RCT (4), control-group design (7), pre-and post-test designs (6), qualitative (20), observational (2) | Patients can enrich medical education by allowing learners to explore patient-centered perspectives in holistic care. For educators this review highlights the lack of an underpinning conceptual basis for which to translate theory into practice. | Lack of robust studies. Measuring outcomes from the perspective of the users. Reporting on the resources directly or indirectly needed to facilitate involvement. | empathy and understanding, communication skills |

| | | | | | | | |
|---|---|---|---|---|---|---|---|
| Happell et al. 2014 | **Consumer involvement in mental health professionals education**<br><br>**Psychiatrists, nurses, psychologists, social workers, and occupational therapists** | NR | Review consumer involvement in the education of mental health professionals | Narrative synthesis of 28 studies: descriptive studies (12), evaluative studies (16) | Consumer involvement in the education of mental health professionals is limited and variable across professions. Evaluations of consumer involvement in 16 courses suggest that students gain insight into consumers' perspectives. | Effect of consumer involvement in education on the behaviors and attitudes of students in healthcare environments | Student perceptions, consumer perceptions, educator perspectives |
| Jha et al. 2009 | **Patient involvement in medical education** | NR | Role and effectiveness of patient involvement in medical education | Narrative synthesis of 47 studies: descriptions of training programs (22), questionnaire surveys (5), interview surveys (5), mixed methods (4), RCT (4), controlled/ comparative studies (2), pre-post design (3), observational designs (1), Delphi design (1) | The majority of studies reported patients in the role of teachers only; others described patient involvement in assessment or curriculum development or in combined roles.<br>The effectiveness of patient involvement was measured by evaluation studies and reported improvements in skills. | Evidence of longer-term impact is lacking. Issues of ethics, psychological impact and influence on education policy are poorly explored. Patient roles, feasibility, cost-effectiveness, and sustainability of programs, as well as positive and negative outcomes are not adequately addressed. | Learner and patient satisfaction/empowerment, skills |
| Lalani et al. 2019 | **Contribution of patient and the public in medical performance processes** | NR | Explore and evaluate the contribution of PPI in medical performance processes to understand its extent, purpose and process | Narrative and inductive synthesis of 48 studies: case series studies (20), cross-sectional (12), qualitative (13), RCT (1), other (2) | Extent of PPI in medical performance processes varies globally and is mainly done through patient feedback or complaints. The emerging evidence suggests that PPI can encourage improvements in the care quality and promote professional development and professionalism. | Need for a better understanding of the actual impact of the different types of PPI in their current format in regulatory processes and systems, as well as potential positive impacts of PPI such as promoting professionalism among doctors and improving the quality of care delivery | Quality of patient care, professional development |
| Murray et al. 2022 | **Patient and public involvement in the development of competency frameworks for health professions** | NR | Determine how patients and the public are involved in the development of competency frameworks for health professions, and whether their involvement influenced the outcome of the competency frameworks | Narrative synthesis of 43 studies: surveys, focus groups, interview studies, Delphi studies, other consensus methods, nominal group technique, workshop and symposium, and other. 15 studies used | Patient and public involvement influenced the competency framework in three different ways: validation or triangulation of competency statements, defining desirable behaviors and attributes, and generating additional competency statements. | Outcomes of PPI in competency framework development, guidance on who, how, when and why patients and the public should be engaged and how to report it | Competency frameworks which include factors of importance to patients and the public |

| | | | | | | | |
|---|---|---|---|---|---|---|---|
| | | | | more than one method. | | | |
| Nguyen et al. 2021 | **Patient involvement in training student pharmacists** | NR | Explore how patients are actively involved in pharmacy education within educational settings, the roles that patients play and the content that they deliver as well as the impact of their involvement | Narrative synthesis of 12 studies: mixed methods (4), qualitative (3), quantitative (5) | Among students: improvements in communication skills, deeper understanding of patients' perspectives, increased confidence in providing care for patients; Among patients: greater satisfaction, empowerment, and knowledge from sharing personal experiences | Long-term impact of patient involvement in pharmacy education, in terms of students' learning outcomes and clinical outcomes among patients | Students' learning outcomes, patient outcomes |
| Reinders et al. 2011 | **Patient feedback interventions as a method of improving practicing physicians' skills** | NR | Review evidence for the effect of feedback from real patients on physicians' general consultation skills | Narrative synthesis of 15 studies: RCT (1), quasi-experimental (5), qualitative (2), cross-sectional (4), cluster-randomized controlled trial (3) | Educational effect on physicians' consultation skills at the four Kirkpatrick levels. Positive results are clustered among studies that used an outcome measure at the low end of Kirkpatrick's hierarchy of educational effect and in qualitative, nonrandomized studies. Only four of the seven studies that assessed level 4 (change in actual performance or results) found a beneficial effect. | High-quality, valid, and reliable tools to assess physicians' consultation performance and high-quality RCTS on effectiveness of the tools. Study the highest level of effect-changes in consultation skills or patient outcomes. Qualitative studies for better understanding of the most effective ways of acquiring and using patient feedback. | Consultation skills, communication skills/behaviour, interpersonal skills, humanistic qualities, nontechnological skills |
| Scott et al. 2020 | **Patient involvement in health advocacy education for postgraduate medical trainees** | NR | To characterize the training approaches that are currently being implemented in postgraduate medical education to teach residents advocacy skills. | Narrative synthesis of 78 studies: qualitative (35), quantitative/semi-quantitative (22), mixed methods (21) | Published interventions varied widely by pedagogical approach and assessment method.<br>Areas of focus included adapting practice to respond to the needs of or advocacy in partnership with patients, communities, or populations served; determinants of health; health promotion; mobilizing resources as needed; and social accountability. | Health advocacy education that target a particular audience, specific theoretical framework(s),<br>novel methods of assessment | Advocacy skills |
