# Supplementary material for: Patient and public involvement in healthcare: a systematic mapping review of systematic reviews – identification of current research and possible directions for future research
Source: BMJ Open. 2024 Sep 19;14(9):e083215. doi: 10.1136/bmjopen-2023-083215 (PMC11418490; doi:10.1136/bmjopen-2023-083215)
Supplement: online supplemental file 4 [file bmjopen-14-9-s004.pdf]

Supplement 4. Patient and public involvement (PPI in systematic review process

(GRIPP2 short-form according to Staniszewska S, Brett J, Simera I, et al. GRIPP2 reporting checklists: tools to improve reporting of patient and public involvement in research. BMJ. 2017:j3453. doi:10.1136/bmj.j3453)

Table 1 Healthcare quality improvement

Publication → ***GRIPP2 ↓***	Bombard et al. 2018	Danhoundo et al. 2018	Evans et al. 2010	Green et al. 2020	Haldane et al. 2019	Kesale et al. 2022	Lloyd et al. 2021	Moore et al. 2019
1. Aim: Report the aim of PPI in the study.	NR	NR	NR	NR	NR	NR	NR	NR
2. Methods: Provide a clear description of the methods used for PPI in the study.	NR	NR	NR	NR	NR	NR	NR	NR
3. Study Results: Outcomes – Report the results of PPI in the study, including both positive and negative outcomes.	NR	NR	NR	NR	NR	NR	NR	NR
4. Discussion and conclusions: Outcomes – Comment on the extent to which PPI influenced the study overall. Describe positive and negative effects.	NR	NR	NR	NR	NR	NR	NR	NR
5. Reflections/critical perspective: Comment critically on the study, reflecting on the things that went well and those that did not, so others can learn from this experience.	NR	NR	NR	NR	NR	NR	NR	NR
Additional info (not part of GRIPP2 short-form):								
Patient or public representative is co-author.	NR	NR	NR	NR	NR	NR	NR	NR
Patient or public representative(s) mentioned in acknowledgements.	NR	NR	NR	NR	NR	NR	NR	Yes

Table 2 **Patient safety**

Publication → <br>***GRIPP2 ↓***	Giap & Park 2021	Lee et al. 2021	Park & Giap 2020
1. Aim:<br>Report the aim of PPI in the study.	NR	NR	NR
2. Methods:<br>Provide a clear description of the methods used for PPI in the study.	NR	NR	NR
3. Study Results:<br>Outcomes – Report the results of PPI in the study, including both positive and negative outcomes.	NR	NR	NR
4. Discussion and conclusions:<br>Outcomes – Comment on the extent to which PPI influenced the study overall. Describe positive and negative effects.	NR	NR	NR
5. Reflections/critical perspective:<br>Comment critically on the study, reflecting on the things that went well and those that did not, so others can learn from this experience.	NR	NR	NR
Additional info (not part of GRIPP2 short-form):			
Patient or public representative is co-author.	NR	NR	NR
Patient or public representative(s) mentioned in acknowledgements.	NR	NR	NR

Table 3 **Community-based initiatives**

Publication → ***GRIPP2 ↓***	Banna & Bersamin 2018	Farnsworth et al. 2014	Haldane et al. 2020	Heintze et al. 2007	Hoon-Chuah et al. 2018	Kerrigan et al. 2013	Moore et al. 2014	Prost et al. 2013	Rass et al. 2020	Sharma et al. 2018
1. Aim: Report the aim of PPI in the study.	NR	NR	NR	NR	NR	NR	NR	NR	NR	NR
2. Methods: Provide a clear description of the methods used for PPI in the study.	NR	NR	NR	NR	NR	NR	NR	NR	NR	NR
3. Study Results: Outcomes – Report the results of PPI in the study, including both positive and negative outcomes.	NR	NR	NR	NR	NR	NR	NR	NR	NR	NR
4. Discussion and conclusions: Outcomes – Comment on the extent to which PPI influenced the study overall. Describe positive and negative effects.	NR	NR	NR	NR	NR	NR	NR	NR	NR	NR
5. Reflections/critical perspective: Comment critically on the study, reflecting on the things that went well and those that did not, so others can learn from this experience.	NR	NR	NR	NR	NR	NR	NR	NR	NR	NR
Additional info (not part of GRIPP2 short-form):										
Patient or public representative is co-author.	NR	Yes (S.H.)	NR	NR	NR	NR	NR	NR	NR	NR
Patient or public representative(s) mentioned in acknowledgements.	NR	Yes	NR	NR	NR	Yes	NR	NR	NR	NR

**Table 4** **Peer-support**						
Publication → ***GRIPP2 ↓***	Gaiser et al. 2021	Genberg et al. 2016	Pitt et al. 2013	Satinsky et al. 2021	Simpson & House 2002	Verma et al. 2022
1. Aim: Report the aim of PPI in the study.	NR	NR	NR	NR	NR	NR
2. Methods: Provide a clear description of the methods used for PPI in the study.	NR	NR	Yes	NR	NR	NR
3. Study Results: Outcomes – Report the results of PPI in the study, including both positive and negative outcomes.	NR	NR	NR	NR	NR	NR
4. Discussion and conclusions: Outcomes – Comment on the extent to which PPI influenced the study overall. Describe positive and negative effects.	NR	NR	NR	NR	NR	NR
5. Reflections/critical perspective: Comment critically on the study, reflecting on the things that went well and those that did not, so others can learn from this experience.	NR	NR	NR	NR	NR	NR
Additional info (not part of GRIPP2 short-form):						
Patient or public representative is co-author.	NR	NR	NR	NR	NR	Yes (P.K.V.)
Patient or public representative(s) mentioned in acknowledgements.	NR	NR	NR	NR	NR	NR

Table 5 **Education of healthcare professionals**

Publication → ***GRIPP2 ↓***	Dijk et al. 2020	Finch et al. 2018	Gordon et al. 2020	Happell et al. 2014	Jha et al. 2009	Lalani et al. 2019	Murray et al. 2022	Nguyen et al. 2021	Reinders et al. 2011	Scott et al. 2020
1. Aim:<br>Report the aim of PPI in the study.	Yes	NR	Yes	NR	NR	NR	NR	NR	NR	NR
2. Methods:<br>Provide a clear description of the methods used for PPI in the study.	Yes	NR	Yes	NR	NR	NR	NR	NR	NR	NR
3. Study Results:<br>Outcomes – Report the results of PPI in the study, including both positive and negative outcomes.	NR	NR	NR	NR	NR	NR	NR	NR	NR	NR
4. Discussion and conclusions:<br>Outcomes – Comment on the extent to which PPI influenced the study overall. Describe positive and negative effects.	NR	NR	NR	NR	NR	NR	NR	NR	NR	NR
5. Reflections/critical perspective:<br>Comment critically on the study, reflecting on the things that went well and those that did not, so others can learn from this experience.	NR	NR	NR	NR	NR	NR	NR	NR	NR	NR
Additional info (not part of GRIPP2 short-form):										
Patient or public representative is co-author.	Yes (M.W.)	NR	Yes, 2 co-authors (E.M. & A.M.)	NR	NR	NR	NR	NR	NR	NR
Patient or public representative(s) mentioned in acknowledgements.	Yes	NR	NR	NR	NR	NR	NR	NR	NR	NR
