# Supplementary material for: Patient and public involvement in healthcare: a systematic mapping review of systematic reviews – identification of current research and possible directions for future research
Source: BMJ Open. 2024 Sep 19;14(9):e083215. doi: 10.1136/bmjopen-2023-083215 (PMC11418490; doi:10.1136/bmjopen-2023-083215)
Supplement: online supplemental file 5 [file bmjopen-14-9-s005.pdf]

Supplement 5.

Health issues covered by number of reviews

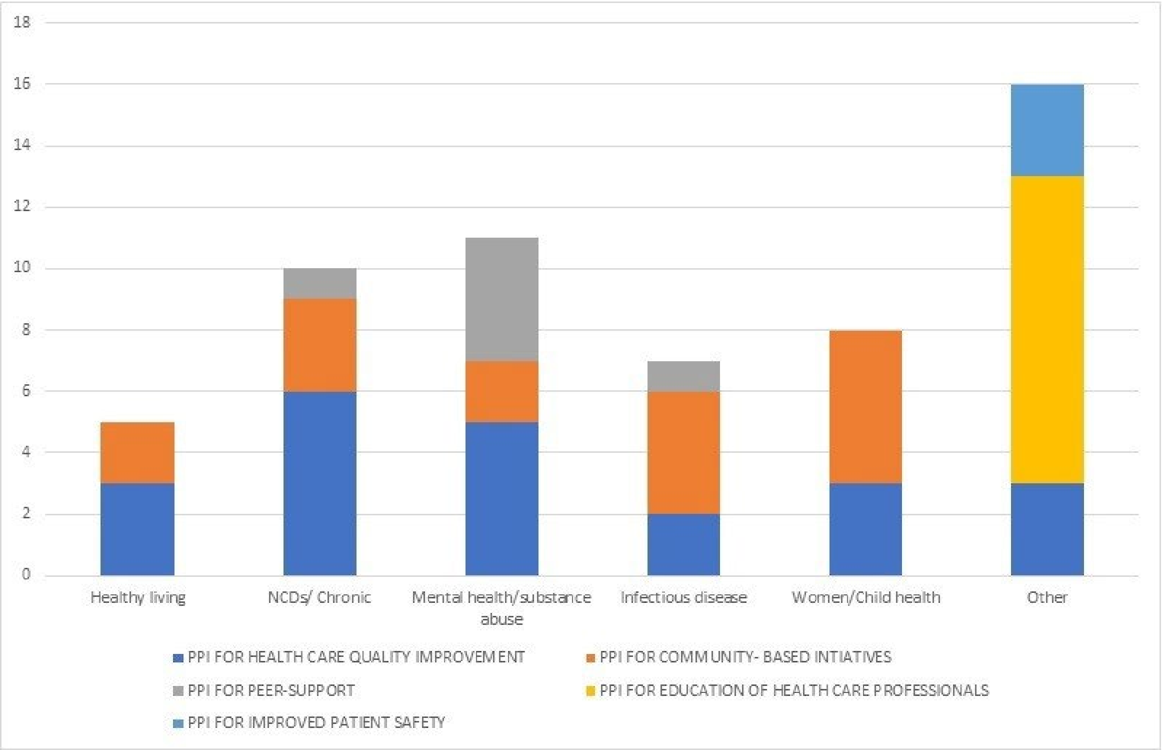

Note: The category *other* includes the quality of healthcare services in general or education of healthcare professionals.

**Number of reviews reporting from each continent**						
**Type of review**	**Europe**	**Oceania**	**North America**	**Africa**	**South America**	**Asia**
EDU	7	7	7		1	4
HCQ	6	4	5	4	3	2
PS	2	2	2			2
PES	4	2	6	3		1
CB	1	2	2	4	4	6

EDU = Education of healthcare professionals, HCQ = Healthcare quality improvement, PS = Patient safety, PES = Peer-support, CB= Community-based initiatives
