# Supplementary material for: Patient and public involvement in healthcare: a systematic mapping review of systematic reviews – identification of current research and possible directions for future research
Source: BMJ Open. 2024 Sep 19;14(9):e083215. doi: 10.1136/bmjopen-2023-083215 (PMC11418490; doi:10.1136/bmjopen-2023-083215)
Supplement: online supplemental file 6 [file bmjopen-14-9-s006.pdf]

PPI for health care quality improvement

- “public involvement” *(Lloyd et al., 2021)*
- “patient engagement”, ”patient involvement” *(Bombard et al., 2018)*
- “participatory approaches” *(Evans et al., 2010)*
- “health service users (HSU)”, “participatory methods” *(Moore et al., 2019)*
- “Experience-based co-design (EBCD)”, “service user” *(Green et al., 2020)*
- “community participation”, “citizen engagement” *(Danhoundo et al., 2018)*
- “community participation”, “community involvement” *(Haldane et al., 2019)*
- “community participation” *(Kesale et al., 2022)*

PPI for improved patient safety

- “Patient and family engagement (PFE)” *(Park and Giap, 2020)*
- “Patient and family involvement (PFI)” *(Giap and Park, 2021)*
- “Involvement of patients and families” and “engagement levels” *(Lee et al., 2021)*

PPI for community-based initiatives

- ”Community involvement”, ”community engagement” *(Banna and Bersamin, 2018; Haldane et al., 2020)*
- “Community engagement”, “community involvement”, “community participation”, “community mobilization” *(Farnsworth et al., 2014)*
- “Community empowerment”, “community involvement”, “community engagement” *(Moore et al., 2014)*
- “Community empowerment interventions” *(Kerrigan et al., 2013)*
- “Community-based interventions”, “community participation” *(Heintze et al., 2007)*
- “Community participation interventions”, “community mobilisation” *(Sharma et al., 2018)*
- “participatory learning and action” *(Prost et al., 2013)*
- “Community participation” *(Rass et al., 2020; Hoon Chuah et al., 2018)*

PPI for peer-support

- “User involvement” *(Simpson and House, 2002)*
- “Consumer-provider” *(Pitt et al., 2013)*
- “Peer provider” *(Gaiser et al., 2021)*
- “Peer-delivered services” *(Satinsky et al., 2021)*
- “Peer interventions” and “Patient engagement” *(Genberg et al., 2016)*
- “Peer coach-led interventions” *(Verma et al., 2022)*

PPI for education of health care professionals

- “Patient involvement” *(Nguyen et al., 2021; Jha et al., 2009; Dijk et al., 2020)*
- “Patient/service user involvement” *(Gordon et al., 2020)*
- “Consumer involvement” *(Happell et al., 2014)*
- “Patient and public involvement”, “engagement methods” *(Murray et al., 2022)*
- “Patient and public involvement (PPI)” *(Lalani et al., 2019)*
- ”Advocacy in partnership with patients, communities, or populations served”, ”involving non–medical community stakeholders” *(Scott et al., 2020)*
- “Patient feedback interventions” *(Reinders et al., 2011)*
- “Patient feedback” *(Finch et al., 2018)*
