# Supplementary material for: Patient and public involvement in healthcare: a systematic mapping review of systematic reviews – identification of current research and possible directions for future research
Source: BMJ Open. 2024 Sep 19;14(9):e083215. doi: 10.1136/bmjopen-2023-083215 (PMC11418490; doi:10.1136/bmjopen-2023-083215)
Supplement: Supplementary file 7 [file bmjopen-2023-083215.reviewer_comments.pdf]

PEER REVIEW HISTORY

BMJ Open publishes all reviews undertaken for accepted manuscripts. Reviewers are asked to complete a checklist review form and are provided with free text boxes to elaborate on their assessment. These free text comments are reproduced below.

ARTICLE DETAILS

**TITLE (PROVISIONAL)**	Patient and public involvement in healthcare: A systematic mapping review of systematic reviews - Identification of current research and possible directions for future research
**AUTHORS**	Bergholtz, Jana; Wolf, Axel; Crine, Vanessa; Cleeve, Helena; Santana, Maria-Jose; Björkman, Ida

VERSION 1 - REVIEW

**REVIEWER NAME**	Talbot, Amelia
**REVIEWER AFFILIATION**	University of Oxford, Nuffield Department of Primary Health Care Sciences
**REVIEWER CONFLICT OF INTEREST**	None
**DATE REVIEW RETURNED**	18-Jan-2024

**GENERAL COMMENTS**	Thank you for submitting your paper to the BMJ Open. This is a systematic mapping review of systematic reviews of patient and public involvement in healthcare. The aim of the review is to provide a summary of current research and identify possible directions for future research. I believe this aim is important given the known importance of patient and public involvement in health care and research. I believe this manuscript would be relevant to the BMJ Open given that they have published on this area before and have a large health following. I have provided some comments below in the order I read the manuscript.<br><br>Methods<br>Patient and public involvement: A strong advantage of this paper is that the team worked with a patient co-researcher. However, I think the information on this section is a bit too detailed. I think the first two sentences would be sufficient.<br>Design: The authors say that conducted a mapping review to provide broad description of a broad research field rather than to produce a synthesis. This method definitely suits their research objectives. I think a sentence on what this means may be needed for clarity. For example, is this mapping what methods were used, what topics were generally explored etc.? The sentence "In relation to PPI in healthcare, the state of research can be mapped while taking into account the abundance and the diversity of research on the topic and provide an overview with intuitive and accessible visualizations" makes no sense to me. I would also recommend the authors check for American spellings. As this is a British journal, I believe spellings should be in British English.

	The Pico criteria is good, but a bit long. It may benefit from being bulleted.<br>The interest in in patient and public involvement. I wonder if this included advisory boards, co-researchers, and other terms that may use to refer to PPI.<br><br>Search Strategy: The authors say that used the PICo framework to upstream the variation in the terminology used for PPI in publications. The use of the term upstream is confusing, and I was not sure what this means. The authors searched three databases, but I wonder if there were any manual searches. Are the two reviewers named on the manuscript? If so who did this reviewing/ screening?<br><br>Inclusion Criteria: The authors use the term “downstream.” I do not know what this means. The authors included English reviews only. Why? The authors also say they focused on journals of patients and public in healthcare. This is confusing as many journals include PPI work but do not focus on this generally.<br><br>Data extraction and analysis: The way the analysis method has been described is confusing and I am unsure if I would be able to follow it myself. The example does not provide any clarification. Was a technology like NVivo used to support analysis?<br><br>Results<br>Characteristics of the included study: The inclusion of the PRISM flow is nice. The description of the studies is clear.<br><br>Results generally: In general, the results seem trustworthy and like they would accurately represent the data shown. The tables are very useful for presenting the data concisely.<br><br>Strengths and limitations<br>For the fourth bullet point, I think the word would be scope not comprehensiveness.<br><br>Introduction<br>The introduction should start with one sentence on what patient and public involvement is before showing the international support.<br>Does your definition of public involvement include charities/ organisations representing patients?<br>The authors do a good job in their title of saying their aim of their review but need to also put this in their introduction.<br>I am unsure whether the rationale for this study is adequate. The authors say that there is debate regarding terms and impact tools. However, the NIHR has clearly provided definitions of what the public and the patient refers to- and I do not really see any debate regarding this.<br>The sentences “In addition, the variety of conceptual frameworks as presented by Greenhalgh et al 2019 provides a starting point for any researcher who is new to involving patients, family caregivers and/or the public. Despite all the effort, there is an urgent need to better understand theoretical underpinnings for PPI in healthcare practice, especially in order to avoid tokenistic efforts.” It is not clear how this would motivate a scoping review of systematic reviews. I think they authors need to think more clearly about why this review is needed.<br><br>Discussion

	"A significant portion" needs to be rephrased as this is not quantitative study.<br>The strengths and limitations section is far too long – especially as there are strengths and limitations at the start. This section is not supposed to cover every strength and limitation but to highlight the main few. I am surprised the authors do not highlight the patient co researcher as a strength.<br><br>The author's contributions seem fair.<br><br>Thank you again for submitting your manuscript. After review, I am going to recommend accept with major revisions for two main reasons: (1) The introduction and rationale for the study need to be made stronger. I am unsure why this systematic review of reviews was needed; and (2) The methods are extremely hard to follow in places and the method of analysis needs to be made clearer to ensure reproducibility. The strengths and limitation section also needs making more concise. The strengths of this manuscript are the inclusion of the patient co-researcher and the tabular results.<br><br>I hope these comments are fair and will help you produce a more well-rounded manuscript. Well done.

**REVIEWER NAME**	Finderup, Jeanette
**REVIEWER AFFILIATION**	Aarhus Universitetshospital, Renal Medicine
**REVIEWER CONFLICT OF INTEREST**	I declare to have no competing interests
**DATE REVIEW RETURNED**	12-Feb-2024

**GENERAL COMMENTS**	Thank you for conducting a mapping review of reviews. In a field with a lot of reviews it is appropriate to conduct a mapping review.<br>A few suggestions for improvements of the paper. It is not clear in the abstract and the background, that that focus is not PPI in healthcare research. This is first stated in the method section, but in the background and in the discussion references about PPI in healthcare research are used. It is not clear that you focus on organisational PPI and exclude individual PPI. This should be more clear in the abstract, background and discussion. You use very few references in the discussion only papers included in your reviews of reviews. I miss a clear definition of PPI and which PPI approach you have chosen.

VERSION 1 – AUTHOR RESPONSE

Reviewer: 1
Ms. Amelia Talbot, University of Oxford
Comments to the Author:
Thank you for submitting your paper to the BMJ Open. This is a systematic mapping review of systematic reviews of patient and public involvement in healthcare. The aim of the review is to provide a summary of current research and identify possible directions for future research. I believe this aim is important given the known importance of patient and public involvement in health care and research. I believe this manuscript would be relevant to the BMJ Open given that they have published on this area before and have a large health following. I have provided some comments below in the order I read the manuscript.

Thank you so much for your kind comments which helped us a lot to improve the manuscript.

1. Methods
Patient and public involvement: A strong advantage of this paper is that the team worked with a patient co-researcher. However, I think the information on this section is a bit too detailed. I think the first two sentences would be sufficient.
The suggested sentences were deleted. However, we added the following to describe our approach better:
A patient co-researcher (JB) was involved in all stages of the review as the first author through a collaborative partnership-focused approach based on Boote et al 2006's principles of successful consumer involvement in research (13,14).

2. Design: The authors say that conducted a mapping review to provide broad description of a broad research field rather than to produce a synthesis. This method definitely suits their research objectives. I think a sentence on what this means may be needed for clarity. For example, is this mapping what methods were used, what topics were generally explored etc.?
We added the following for clarity:
Design
A mapping review aims to provide a broad overview of a ~~description of a broad~~ research field, categorising existing literature to identify gaps from which further reviews and/or primary research can be commissioned (15).

Regarding methods used, please refer to our answer of point 12.
The topics generally explored in our mapping review are those stated in our research questions:
PURPOSE
The overarching goal of this mapping review is to ~~identify and~~ provide an overview of ~~research on~~ PPI in the meso- and macro-level of healthcare ~~practices~~ and identify possible directions for future research. Our review addresses the following research questions:

1. In which contexts is PPI conducted~~ in healthcare practice~~?
2. What are the characteristics of participants involved in PPI~~ in healthcare practice~~?
3. Which conceptualis~~z~~ations (terms, frameworks) of PPI are used~~ in healthcare practice~~?
4. Which outcome measures are reported for PPI~~ in healthcare practice~~?
5. What findings and research gaps can be identified~~ for PPI in healthcare practice~~?

3. The sentence "In relation to PPI in healthcare, the state of research can be mapped while taking into account the abundance and the diversity of research on the topic and provide an overview with intuitive and accessible visualizations" makes no sense to me.
This sentence has been deleted.

4. I would also recommend the authors check for American spellings. As this is a British journal, I believe spellings should be in British English.
We have checked the manuscript for American spellings and adjusted the following words:
standardiz → standardis**
organiz → organis**
prioritize → prioritise
conceptualiz → conceptualis**
summarized → summarised
thematization → thematisation
categoriz → categoris**
mobilization → mobilisation
decentralization → decentralisation
theorized → theorised

5. The Pico criteria is good, but a bit long. It may benefit from being bulleted.
This has been adjusted:
The generally explored topics trace back to our ~~The~~ research questions, which were defined in accordance with the PICo framework (**P**opulation or Problem, **I**nterest and **Co**ntext) for qualitative studies (18) with:
~~. The ***population*** includes patients and public of all ages involved in PPI initiatives or interventions in healthcare practice. This population was not restricted to any socio-demographic characteristic, a~~

geographical region, a particular healthcare service type or a particular healthcare sector. In this review, the ***interest*** is the involvement of patients and the public in healthcare practice and how they can be change-agents to improve other people's healthcare. We use involvement as an umbrella term to refer to all processes embracing the participation of patients and public to improve healthcare at the system level. Reviews focused on patients' participation in their own individual healthcare decisions or on their experience of living with a certain condition were excluded. By ***context*** we mean healthcare contexts in which PPI is implemented.

- ***Population:*** patients and public of all ages involved in PPI initiatives or interventions in healthcare practice, not restricted to any socio-demographic characteristic or geographical region,
- ***Interest:*** the involvement of patients and the public in the meso- and macro-level of healthcare,
- ***Context:*** all healthcare contexts in which PPI is implemented, not restricted to any particular healthcare service type or a particular healthcare sector.

6. The interest in in patient and public involvement. I wonder if this included advisory boards, co-researchers, and other terms that may use to refer to PPI.
Yes, this includes advisory boards – however, it does not include co-researchers (see answer to point 17). We did not map all the various methods used for PPI in healthcare at meso- and macro-level; this could also be a potential topic for a further review.

7. Search Strategy: The authors say that used the PICo framework to upstream the variation in the terminology used for PPI in publications. The use of the term upstream is confusing, and I was not sure what this means.
We deleted the words upstream and downstream (see point 9) as we understand this may be confusing. We intended to use it to describe steps before and after conducting the search with before (upstream) deciding on PICo framework and after (downstream) deciding on inclusion/exclusion criteria.

8. The authors searched three databases, but I wonder if there were any manual searches. Are the two reviewers named on the manuscript? If so who did this reviewing/ screening?
We did conduct manual searches to validate our search strategy. Yes, the reviewers are named on the manuscript, and we added the initials now:
The screening of abstracts and titles was performed independently by two reviewers (first search: VC and JB, updated search: IB and JB). Disagreements were resolved through discussion or by consulting an additional review author (AW).

9. Inclusion Criteria: The authors use the term "downstream." I do not know what this means.
See response to point 7.

10. The authors included English reviews only. Why?
We assume that most peer-reviewed systematic reviews are typically published in English language while they may cover single studies in other languages. For example, the following systematic reviews without language restrictions: Haldane et al. 2019, Haldane et al. 2020, Kerrigan et al. 2013, and Sharma et al. 2018 were included in our study.

11. The authors also say they focused on journals of patients and public in healthcare. This is confusing as many journals include PPI work but do not focus on this generally.
This is probably a misunderstanding as we meant to say:
Systematic reviews of empirical studies, which focused on the involvement of patients and the public at the meso- and macro-level in healthcare, were eligible for inclusion when published in English in peer-reviewed journals ~~focused on the involvement of patients and the public in healthcare to improve care practices were eligible for inclusion~~.

12. Data extraction and analysis: The way the analysis method has been described is confusing and I am unsure if I would be able to follow it myself. The example does not provide any clarification. Was a technology like NVivo used to support analysis?
We used Rayyan for screening, but data extraction was conducted with Excel®. We adjusted the text in the section on Data extraction and thematisation to better describe our analysis methods (We hope that it is clearer now):
Data extraction and thematisation
A standardi~~z~~sed data extraction form was used to extract data in Excel® (supplemental file 1). This form was predeveloped, pilot tested on five randomly selected studies (by JB, VC and HC) and then adapted before the overall data extraction process started. Data was extracted ~~(~~by JB, VC and IB~~)~~ ~~following the PICo framework and research questions~~. We then systematically ~~searched, identified, and~~ summaris~~z~~ed extracted data ~~existing literature~~ related to our research questions and organised results in narrative, visual or tabular formats to provide the reader with a clear and structured overview.
More specifically, for research questions 1 (In which contexts is PPI conducted?) and 4 (Which outcome measures are reported for PPI?), ~~First,~~ extracted data was ~~first categorized and~~ organis~~z~~ed in related themes~~by inductively creating thematic categories~~. ~~The categories captured key aspects of the research questions and facilitated data structuring.~~ Preliminary themes for context and outcome categories were formed which were refined again and again as the extracted data were re-examined in an iterative process with continuing discussion between IB and JB. ~~As an example, regarding~~ For outcomes, we included any proposed or eventuating positive or negative change short- or long term.
For research question 3 (Which conceptualisations (terms, frameworks) of PPI are used?), in addition to data extraction, frameworks and guidance documents cited in the reviews were tracked down to the original publication and collected. To capture the broad terminology used in the reviews, the pdf-file of each article was assessed via the search function to count which term(s) and/or combination of terms were used most often to describe and discuss PPI.
~~The results were then organized in narrative, visual or tabular formats to provide the reader with a clear and structured overview.~~
Results
Characteristics of the included study: The inclusion of the PRISM flow is nice. The description of the studies is clear. Results generally: In general, the results seem trustworthy and like they would accurately represent the data shown. The tables are very useful for presenting the data concisely.
Thank you so much for your kind comments.

13. Strengths and limitations
For the fourth bullet point, I think the word would be scope not comprehensiveness.
Yes, this has been adjusted:
• Grey literature was excluded, and language was restricted to English, potentially limiting the scope of this mapping review.

Introduction
14. The introduction should start with one sentence on what patient and public involvement is before showing the international support.
We added the following sentence:
Patient and public involvement (PPI) in healthcare has been broadly defined as "ways in which patients can draw on their experience and members of the public can apply their priorities to the evaluation, development, organisation and delivery of health services" (1).

15. Does your definition of public involvement include charities/ organisations representing patients?
Yes, it does. We added (at the end of the introduction):
While being attentive to the distinctions that can be drawn, we use 'patient and public involvement' to include patients, survivors, family members, informal caregivers, service users, communityi~~ies~~ or charity representatives, and citizens.

16. The authors do a good job in their title of saying their aim of their review but need to also put this in their introduction.
We added this in the purpose section (below the introduction):
PURPOSE
The overarching goal of this mapping review is to ~~identify and~~ provide an overview of ~~research on~~ PPI in the meso- and macro-level of healthcare ~~practices~~ and identify possible directions for future research.

and in the abstract as well:
ABSTRACT
Objectives To provide an overview of patient and public involvement (PPI) in the meso- and macro-level of healthcare (different from PPI in research) and identify directions for future research by mapping contexts, terminology, conceptual frameworks, measured outcomes, and research gaps.

17. I am unsure whether the rationale for this study is adequate. The authors say that there is debate regarding terms and impact tools. However, the NIHR has clearly provided definitions of what the public and the patient refers to- and I do not really see any debate regarding this. The sentences *"In addition, the variety of conceptual frameworks as presented by Greenhalgh et al 2019 provides a starting point for any researcher who is new to involving patients, family caregivers and/or the public. Despite all the effort, there is an urgent need to better understand theoretical underpinnings for PPI in healthcare practice, especially in order to avoid tokenistic efforts."* It is not clear how this would motivate a scoping review of systematic reviews. I think they authors need to think more clearly about why this review is needed.
We changed the background section substantially (see below), putting an emphasis on the distinctions that need to be drawn between PPI in research vs. PPI in healthcare as well as for patient involvement in individual care vs. PPI at the meso- and macro-level (e.g., Modigh et al. 2021). Our mapping review aims to cover PPI at the meso- and macro-level in healthcare.

Another justification for our study (and we know this from own experience) is that there is much frustration over the lack of guidance on how to achieve meaningful and impactful PPI at the meso- and macro-level in healthcare. We think that conceptual frameworks such as those compiled by Greenhalgh et al. 2019 for PPI in research are helpful for beginners as a starting point, however, no such compilation exists so far to our knowledge for PPI at the meso- and macro-level in healthcare. We hope that our compilation of frameworks and guidance (Table 2) can serve as starting point for beginners as well as for additional studies.

BACKGROUND

Patient and public involvement (PPI) in healthcare has been broadly defined as “ways in which patients can draw on their experience and members of the public can apply their priorities to the evaluation, development, organisation and delivery of health services” (1). Several international and national policy documents support the principles of ~~patient and public involvement (~~PPI~~)~~ in healthcare (2,3). An early example is the 1978 Declaration of Alma-Ata which stated the community as central to development of healthcare services (4). In Europe, the implementation of person-centred care can be fostered with the standard EN 17398:2020 *Patient involvement in healthcare – Minimum requirements for person-centred care* (5). General arguments for PPI are based on democratic and ethical rights (6)~~7~~, ~~increased knowledge production (8),~~ as well as patient empowerment (7).

Despite the international interest in enabling healthcare systems ~~and researchers~~ to involve patients, family caregivers, communities and the public in decisions, ~~there are~~ major challenges for studying the impact and effects of PPI are inconsistent terminology, methods and reporting (8,9). Even though some of the core aspects may overlap, Modigh et al 2021 pointed out that it is important to distinguish between PPI in healthcare versus PPI in research in order to develop a more nuanced understanding (9). In their scoping review of reviews, they found many reviews reporting on the impact of patient involvement in individual care and very few reviews covering PPI at the meso- and macro-level of healthcare (9). This is surprising, especially because this field has seen a great deal of activity in the past two decades.

Several studies also raise the lack of a theoretical basis for understanding how PPI induces improvement as a major problem for PPI in practice (10,11). There is a commonly expressed frustration in the field regarding the difficulty in describing how to do PPI, i.e. without engaging in tokenism, so it will achieve meaningful results (11,12). ~~For instance, the variety of terms with considerable overlap (10-12), various opinions related to its impact and measuring it (14,15) and ongoing discussions on the differences between *‘patient involvement’* and *‘public involvement’* (16,17). In addition, the variety of conceptual frameworks as presented by~~ While Greenhalgh et al 2019 provide a starting point for anyone~~ researcher~~ who is new to ~~involving patients, family caregivers and/or the public~~ PPI in research (13) – to our knowledge, such a collection of conceptual frameworks is lacking for PPI at the meso- and macro-level of healthcare. ~~Despite all the effort, there is an urgent need to better understand theoretical underpinnings for PPI in healthcare practice, especially in order to avoid tokenistic efforts (19).~~

With our systematic mapping review of reviews, we aim to provide a broad overview of activities in the field of PPI at the meso- and macro-level in healthcare while addressing the variation in terminology. ~~address some of these challenges by providing an overview of how PPI in healthcare practice has been conceptualized and which outcomes have been measured within contexts and settings where patients, family carers and the public become change-agents to improve other people’s healthcare.~~ While being attentive to the distinctions that can be drawn, we use ‘*patient and public involvement’* to include patients, survivors, family members, informal caregivers, service users, community~~ies~~ or charity representatives, and citizens.

Discussion

18. “A significant portion” needs to be rephrased as this is not quantitative study.

This was changed to “many”.

19. The strengths and limitations section is far too long – especially as there are strengths and limitations at the start. This section is not supposed to cover every strength and limitation but to highlight the main few. I am surprised the authors do not highlight the patient co researcher as a strength.

The strengths and limitations section was shortened significantly. Inclusion of the patient co-researcher is now highlighted as a strength of this manuscript.

STRENGTHS AND LIMITATIONS

This study benefited from the inclusion of a patient co-researcher (JB) as team member and author, providing a patient perspective throughout the whole process. We ~~This systematic mapping review~~

employed a comprehensive search strategy ~~based on the PICo framework~~, allowing for a wide exploration of the terminology used in publications related to patient and public involvement (PPI) in healthcare ~~practice~~. ~~This approach ensured that a diverse range of relevant literature was captured.~~ A rigorous screening process was conducted, ~~resulting in the inclusion of 37 systematic reviews that met specific eligibility criteria. This~~ and the careful selection process enhances the reliability and relevance of the included reviews. ~~However, aiming to capture a broad range of terminology used for PPI, the heterogeneity of terminology in this field makes it challenging to ensure the inclusivity of all relevant publications. Some studies may use unique or less common terms that were not captured in the search strategy.~~ A~~nother~~ limitation is that the language was restricted to English, which may introduce language bias where relevant research published in other languages may have been excluded, potentially limiting the ~~comprehensiveness~~ scope of the mapping review. Also, grey literature was excluded, and such can sometimes contain valuable insights not found in peer-reviewed journals, and its exclusion may lead to the omission of relevant information. ~~The review employed an iterative process for data extraction and thematization, involving ongoing discussions between researchers. This approach enhances the depth and accuracy of data analysis, ensuring that key aspects related to PPI were captured. A standardized data extraction form was used, predeveloped, and pilot-tested to ensure consistency and completeness in the data extraction process. This standardized approach minimizes the risk of bias in data collection.~~
The study protocol was registered on the Open Science Forum, providing transparency about the research process, and facilitating reproducibility. The method for systematic mapping review was chosen as this is useful for identifying patterns in a very large body of literature. Mapping reviews, by design, do not typically include quality assessment or statistical analyses of the included reviews (15). While this approach provides a broad overview of research activity, it does not evaluate the methodological quality or rigor of the reviews themselves and does not allow for an in-depth description of the field. However, a strength of our mapping review is that key data and findings are displayed in tabular format.
We did not check whether reviews included the same references. Some reviews studied similar topics and may therefore have included some of the same studies.

20. The author's contributions seem fair.
Thank you again for submitting your manuscript. After review, I am going to recommend accept with major revisions for two main reasons: (1) The introduction and rationale for the study need to be made stronger. I am unsure why this systematic review of reviews was needed; and (2) The methods are extremely hard to follow in places and the method of analysis needs to be made clearer to ensure reproducibility. The strengths and limitation section also needs making more concise. The strengths of this manuscript are the inclusion of the patient co-researcher and the tabular results.
I hope these comments are fair and will help you produce a more well-rounded manuscript. Well done.
Tabular results were included in the strengths of this manuscript (see text under point 19).
Thank you so much! We appreciate your thoughtful comments. They helped us a lot to improve the manuscript.

Response to Reviewer 2

Reviewer: 2
Dr. Jeanette Finderup, Aarhus Universitetshospital
Comments to the Author:
Thank you for conducting a mapping review of reviews. In a field with a lot of reviews it is appropriate to conduct a mapping review.
Thank you so much for your kind comments which helped us a lot to improve the manuscript.

1. A few suggestions for improvements of the paper. It is not clear in the abstract and the background, that that focus is not PPI in healthcare research. This is first stated in the method section, …
We added this in the abstract and changed the background section significantly. We hope that it is much clearer now.

ABSTRACT

Objectives To provide an overview of patient and public involvement (PPI) in the meso- and macro-level of healthcare (different from PPI in research) and identify directions for future research by mapping contexts, terminology, conceptual frameworks, measured outcomes, and research gaps.

BACKGROUND
Patient and public involvement (PPI) in healthcare has been broadly defined as "ways in which patients can draw on their experience and members of the public can apply their priorities to the evaluation, development, organisation and delivery of health services" (1). Several international and national policy documents support the principles of ~~patient and public involvement (~~PPI~~)~~ in healthcare (2,3). An early example is the 1978 Declaration of Alma-Ata which stated the community as central to development of healthcare services (4). In Europe, the implementation of person-centred care can be fostered with the standard EN 17398:2020 *Patient involvement in healthcare – Minimum requirements for person-centred care* (5). General arguments for PPI are based on democratic and ethical rights (6)~~7~~, ~~increased knowledge production (8),~~ as well as patient empowerment (7).

Despite the international interest in enabling healthcare systems ~~and researchers~~ to involve patients, family caregivers, communities and the public in decisions, ~~there are~~ major challenges for studying the impact and effects of PPI are inconsistent terminology, methods and reporting (8,9). Even though some of the core aspects may overlap, Modigh et al 2021 pointed out that it is important to distinguish between PPI in healthcare versus PPI in research in order to develop a more nuanced understanding (9). In their scoping review of reviews, they found many reviews reporting on the impact of patient involvement in individual care and very few reviews covering PPI at the meso- and macro-level of healthcare (9). This is surprising, especially because this field has seen a great deal of activity in the past two decades.

Several studies also raise the lack of a theoretical basis for understanding how PPI induces improvement as a major problem for PPI in practice (10,11). There is a commonly expressed frustration in the field regarding the difficulty in describing how to do PPI, i.e. without engaging in tokenism, so it will achieve meaningful results (11,12). ~~For instance, the variety of terms with considerable overlap (10-12), various opinions related to its impact and measuring it (14,15) and ongoing discussions on the differences between *'patient involvement'* and *'public involvement'* (16,17). In addition, the variety of conceptual frameworks as presented by~~ While Greenhalgh et al 2019 provide a starting point for anyone~~ researcher~~ who is new to ~~involving patients, family caregivers and/or the public~~ PPI in research (13) – to our knowledge, such a collection of conceptual frameworks is lacking for PPI at the meso- and macro-level of healthcare. ~~Despite all the effort, there is an urgent need to better understand theoretical underpinnings for PPI in healthcare practice, especially in order to avoid tokenistic efforts (19).~~

With our systematic mapping review of reviews, we aim to provide a broad overview of activities in the field of PPI at the meso- and macro-level in healthcare while addressing the variation in terminology. ~~address some of these challenges by providing an overview of how PPI in healthcare practice has been conceptualized and which outcomes have been measured within contexts and settings where patients, family carers and the public become change-agents to improve other people's healthcare.~~ While being attentive to the distinctions that can be drawn, we use '*patient and public involvement'* to

include patients, survivors, family members, informal caregivers, service users, community~~ies~~ or charity representatives, and citizens.

2. … but in the background and in the discussion references about PPI in healthcare research are used.
We now use very few references for PPI in research. Referring to the study of Modigh et al. 2021 (new reference) helps us to distinguish between PPI in research vs. PPI in healthcare. We kept Greenhalgh et al.'s 2019 article on frameworks for PPI in research as reference - we find it important to cite keeping in mind readers who might be new to PPI in general.

3. It is not clear that you focus on organisational PPI and exclude individual PPI. This should be more clear in the abstract, background and discussion.
We made this clearer in the abstract, background and discussion by referring to PPI in the meso- and macro-level of healthcare.

4. You use very few references in the discussion only papers included in your reviews of reviews.
We added the following references in the discussion:

2. World Health Organization. WHO global strategy on people-centred and integrated health services: interim report. [Internet]. 2015. 48 p. Available from: https://www.afro.who.int/sites/default/files/2017-07/who-global-strategy-on-pcihs-main-document_final.pdf

10. Usher S, Denis JL. Exploring expectations and assumptions in the public and patient engagement literature: A meta-narrative review. Patient Educ Couns. 2022 Aug 1;105(8):2683–92.

11. Matthews R, Kaur M, French C, Baker A, Reed J. How helpful are Patient and Public Involvement strategic documents - Results of a framework analysis using 4Pi National Involvement Standards. Res Involv Engagem. 2019 Nov 4;5(1):31.

95. Masterson D, Areskoug Josefsson K, Robert G, Nylander E, Kjellström S. Mapping definitions of co-production and co-design in health and social care: A systematic scoping review providing lessons for the future. Health Expect. 2022;25(3):902–13.

102. Ocloo J, Garfield S, Franklin BD, Dawson S. Exploring the theory, barriers and enablers for patient and public involvement across health, social care and patient safety: a systematic review of reviews. Health Res Policy Syst. 2021 Jan 20;19(1):8.

DISCUSSION

…

A notable shift in the publication of systematic reviews can be seen, with a growing interest in PPI in healthcare ~~practice~~ after 2015. This trend reflects the increasing recognition of the importance of involving patients and the public in healthcare decision-making processes (2,10,95).

…

The data reveal a wide range of terms used to describe and discuss PPI at the meso- and macro-level of healthcare. This diversity in terminology can be both enriching and challenging, as it reflects the evolving nature of the field (10,95).

….

To measure effects and impact of PPI it is also crucial to consider the complex- interplay of various factors leading to a certain outcome (11)

…

Our mapping review of systematic reviews complements recently published studies in several ways. Usher and Denis 2022 explored storylines in the PPI literature in a meta-narrative review to better understand persistent difficulties in the transformation of healthcare systems (10). They concluded that developments across micro-, meso- and macro-levels need to be captured to see how they support one another to drive, enable and sustain change. The systematic review of reviews by Ocloo et al 2021 explored theory, barriers and enablers for PPI across health, social care and patient safety (102) and found that the development of theory-driven approaches is a neglected area. Greenhalgh et al 2019 reported on frameworks for PPI in research (13), whereas we cover frameworks and

conceptual guidance for PPI in the meso- and macro-level of healthcare (see Table 2) which can be helpful for those who are new to engaging patients and the public.

5. I miss a clear definition of PPI and which PPI approach you have chosen.
We have added Tritter's (2009) definition of PPI in healthcare in the beginning of the background section and clarified in the background section that we focus on PPI in the meso- and macro-level of healthcare (see text under point 1).

When it comes to the PPI approach we have chosen for including a patient co-researcher, we clarified in the beginning of the method section:
A patient co-researcher (JB) was involved in all stages of the review as the first author through a collaborative partnership-focused approach based on Boote et al 2006's principles of successful consumer involvement in research (13,14).

VERSION 2 - REVIEW

**REVIEWER NAME**	Finderup, Jeanette
**REVIEWER AFFILIATION**	Aarhus Universitetshospital, Renal Medicine
**REVIEWER CONFLICT OF INTEREST**	I declare that I have not competing interests
**DATE REVIEW RETURNED**	18-Apr-2024

**GENERAL COMMENTS**	From my point of view, you have addressed the comments from editor, the other reviewer and from me. You have made it more clear that your focus is on PPI in a meso and macro level and not in research and not on micro level. But adding the time of search revealed that your search needs to be updated to be less than a year old.

VERSION 2 – AUTHOR RESPONSE

Thank you so much for your comments on the revision of our manuscript.

We do not have the capacity to update the search a second time. We highlighted this in the limitations section (bullet points after abstract).
